# The cellular basis of cartilage growth and shape change in larval and metamorphosing *Xenopus* frogs

**DOI:** 10.1371/journal.pone.0277110

**Published:** 2023-01-12

**Authors:** Christopher S. Rose

**Affiliations:** Department of Biology, James Madison University, Harrisonburg, Virginia, United States of America; University of Colorado Boulder, UNITED STATES

## Abstract

As the first and sometimes only skeletal tissue to appear, cartilage plays a fundamental role in the development and evolution of vertebrate body shapes. This is especially true for amphibians whose largely cartilaginous feeding skeleton exhibits unparalleled ontogenetic and phylogenetic diversification as a consequence of metamorphosis. Fully understanding the evolutionary history, evolvability and regenerative potential of cartilage requires in-depth analysis of how chondrocytes drive growth and shape change. This study is a cell-level description of the larval growth and postembryonic shape change of major cartilages of the feeding skeleton of a metamorphosing amphibian. Histology and immunohistochemistry are used to describe and quantify patterns and trends in chondrocyte size, shape, division, death, and arrangement, and in percent matrix from hatchling to froglet for the lower jaw, hyoid and branchial arch cartilages of *Xenopus laevis*. The results are interpreted and integrated into programs of cell behaviors that account for the larval growth and histology, and metamorphic remodeling of each element. These programs provide a baseline for investigating hormone-mediated remodeling, cartilage regeneration, and intrinsic shape regulating mechanisms. These programs also contain four features not previously described in vertebrates: hypertrophied chondrocytes being rejuvenated by rapid cell cycling to a prechondrogenic size and shape; chondrocytes dividing and rearranging to reshape a cartilage; cartilage that lacks a perichondrium and grows at single-cell dimensions; and an adult cartilage forming *de novo* in the center of a resorbing larval one. Also, the unexpected superimposition of cell behaviors for shape change onto ones for larval growth and the unprecedented exploitation of very large and small cell sizes provide new directions for investigating the development and evolution of skeletal shape and metamorphic ontogenies.

## Introduction

Cartilage precedes bone in both the development and evolution of vertebrate bodies. Cartilage and cartilage-like tissues are widespread in invertebrates [1], and cartilage is the first and sometimes only skeletal tissue to appear in vertebrates, forming a template for building much of the adult skeleton. Its early appearance means that cartilage predetermines many functionally and phylogenetically important spatial relationships involving the shape and arrangement of endochondral and dermal bones as well as muscles, nerves, and blood vessels [2–5]. Further, the extent to which the cartilage skeleton is subsequently replaced and augmented by endochondral and dermal bone varies considerably across vertebrates [2, 6, 7]. Some clades, e.g., hagfish, lampreys, chondrichthyans, sturgeons, and paddlefish, remain entirely or largely cartilaginous, and all others retain different amounts of cartilage in their adult skeletons. How much cartilage is retained becomes important when one recognizes that cartilage and bone are bound to different cell behaviors for growth. Whereas the mineralized matrix of bone permits only the accretion and removal of cells and matrix on its surface, cartilage additionally uses internal or interstitial cell behaviors like cell division, rearrangement, death, and growth to grow and change shape [8–10]. Such mechanistic differences are thought to impact how adult skeletons have diversified across vertebrates [11, 12].

Our understanding of cartilage formation comes largely from mammals and birds, and from three foci of study: the embryonic origins of the cells that become cartilage [13, 14]; the transcription factors, signaling proteins and other factors that control their migration, condensation and differentiation into skeletal rudiments [7, 15–20]; and how the rudiments either expand and coalesce to produce largely contiguous assemblages like the neurocranium [21, 22], or separate into discrete elements like those of the viscerocranium [23–25] and limb skeleton [26–28]. Research on cartilage growth and its role in regulating skeletal shape focuses largely on growth plates [29] and synchondroses [21]. Growth plates are regions of cartilage that are maintained postembryonically at the ends of long bones and that support bone elongation prior to being replaced by bone. They occur in the limb bones of all tetrapods [30–36], and in branchial arch elements of teleost fish [33, 37]. Synchondroses, which are effectively pairs of opposing growth plates, are known only in amniotes, occurring in the floor of the skull, between the centra and neural arches of vertebrae, and between elements of the pelvis bone [19, 21, 22, 38]. What these growth features have in common is regionalized and directional cell behaviors, meaning that cell division, rearrangement, apposition, matrix secretion, growth and/or death are confined to distinct zones, and that planes of cell division, apposition and intercalation are aligned to produce growth in specific directions. Evolutionary changes in the feedback loops that regulate these cell behaviors can thus produce dramatic changes in limb bone length, as seen in bats, jerboas and short-legged dogs [39–41].

This amniote-centered view of cartilage growth as highly regionalized and directional cell behaviors occurring in advance of bone replacement leaves unanswered several fundamental questions about how cartilage grows and evolves in the absence of endochondral replacement. How widespread are regionalized cell behaviors in nonamniotes, how does cartilage grow in the absence of endochondral replacement, and how is its shape regulated throughout life? More generally, how might the cell behaviors used in cartilage growth impact its histology and function, as well as its evolutionary potential or evolvability for shape change, and its capacity for regeneration?

Amphibians are especially relevant because their feeding and breathing skeleton (viscerocranium) persists largely as cartilage and exhibits both simple and complex shapes that differ among species, grow with little or no shape change in larvae and adults, and undergo abrupt and radical shape changes during a midlife metamorphosis [11, 12, 42, 43]. The amphibian larval viscerocranium is formed almost entirely from neural crest cells that migrate into pharyngeal arches, and condense and differentiate into a flexible and partially moveable network of cartilaginous rods, bars and plates. The first, second and third arch skeletons are all distinctly shaped, and their large ventral elements, meaning the lower jaw (Meckel’s cartilage and in frogs an infrarostral), hyoid (ceratohyal), and branchial arch (ceratobranchial) cartilages, can exhibit dramatic departures from simple rod or bar shapes. The viscerocranium is remodeled at metamorphosis to eliminate the elements used for gill support, and to convert from suction feeding to tongue protrusion and biting and engulfing prey. The larval shapes and the shape changes at metamorphosis vary significantly between and within frogs and salamanders, and with profound effects on feeding style [44, 45].

Based on morphological and histological descriptions of cartilage growth and metamorphosis in frogs and salamanders, Rose [11, 12] proposed that amphibians employ different programs of cell division, cell death, cell growth, and matrix secretion to grow larval cartilages with different shapes and histologies, and to change their shapes at metamorphosis. Three specific programs were proposed to drive shape change at metamorphosis: 1) de novo condensations to add new parts onto existing larval cartilages, 2) reshaping and resizing larval cartilages by spatially integrated mixes of cell division, cell death and matrix secretion, and 3) reshaping and resizing larval cartilages by spatially segregated mixes of cell division, cell death and matrix secretion.

This study tests these proposals by describing and quantifying cell division, cell death, changes in cell size, shape and orientation, and matrix accumulation for the lower jaw, ceratohyal, and ceratobranchials of the frog *Xenopus laevis* from one day after hatching to one week past the end of metamorphosis. These three cartilages are particularly well suited for this analysis since they have distinct larval shapes, histologies, and functions in larval and postmetamorphic life, and their size and shape changes during larval and postmetamorphic growth and metamorphosis have been carefully documented in bivariate and multivariate analyses [42]. Also, hormone induction experiments show that whereas their larval growth is not dependent on thyroid hormone (TH), their metamorphic shape changes are TH-mediated and the amount of shape change that can be induced varies with thyroid hormone type (T4 or T3), concentration, and larval stage [46]. The size and shape changes that occur naturally and in induced specimens are investigated here using labels and metrics for cell division, cell death, cell size, shape and orientation, and matrix accumulation. The temporal and spatial patterns of these features are used to infer the programs of cell behaviors that drive the larval growth and shape change of each cartilage.

## Materials and methods

### Ethics statement

All live animal procedures were carried out with written consent from the James Madison University Institutional Animal Care and Use Committee (protocols #A05-12, A10-15, A18-01, and 20–1569), and in accordance with the National Research Council Guide for the Care and Use of Laboratory Animals (8^th^ edition).

### Live animals

*Xenopus laevis* offspring were produced from hormone-injected adults, and housed, raised, fed, and euthanized using previously published materials and methods [42, 46, 47]. Specimens were staged using the Nieuwkoop Faber (NF) system [48], and within-stage variation in age and body size was minimized by raising animals under optimal growth conditions [47] and sampling only fast growers and developers. Although forelimb eruption at NF 58 is generally considered the start of frog metamorphosis, NF 59–66 are the stages spanning metamorphic shape change in the three cartilages of interest here; NF 66+ and NF 67 refer respectively to froglets that are 1–3 days and 1 week past NF 66 (complete tail loss).

Bromodeoxyuridine (BrdU), which labels cells in S phase, was applied as a 24-hr incubation and a 2-hr pulse. BrdU-incubated tadpoles < NF 55 were immersed for 24 hours in 0.05% BrdU and fixed. BrdU-incubated tadpoles ≥ NF 55 were injected intraperitoneally with, depending on their size, 4–8 μl of 0.3% BrdU, and fixed 24 hr later. For BrdU pulse treatments, NF 55–56 and NF 58–9 tadpoles were injected with 6 μl of 0.3% BrdU and 2 hr later with 4 μl of 1% thymidine. They were then raised in 0.05% thymidine for 5 days, moved to normal rearing solution afterwards, and euthanized 1–5 and 18 days after injection. T3 induction treatments followed previously published techniques [46].

### Histology, immunohistochemistry, and whole mount staining

All BrdU-exposed specimens were fixed in 2% trichloroacetic acid (TCA) for 2 hr at 22°C (room temperature or RT) or 100% methanol for 1 hr at RT [49]. Specimens intended for caspase antibody labeling were fixed in Dent’s fixative (80% methanol, 20% dimethyl sulfoxide) for 2 hr at RT. All others were fixed in 10% neutral buffered formalin (NBF) for 24 hr at RT, and, if not dehydrated within the next 24 hr, were stored for a maximum of 1 week in 70% ethanol at 4°C.

NBF- and TCA-fixed heads were dehydrated in an ethanol series, cleared in xylene, embedded in Paraplast Plus wax, and sectioned frontally or transversely at 10 μm; methanol and Dent’s fixed tissue were dehydrated in a second methanol before being moved to wax. The primary antibodies used are mouse G3G4 for BrdU (1:200, ascites fluid, or 1:100 concentrate, Developmental Studies Hybridoma Bank), mouse monoclonal anti-proliferating cell nuclear antigen (1:3000, aPCNA, P-8825, Sigma), rabbit anti-active Caspase-3 (1:250, Cat. # 559565, BD Pharmingen), rabbit cleaved caspase-3 (1:200, Asp175, #9661, Cell Signaling); and mouse 5D3 for E-cadherin and II-II6B3 for collagen (both 1:200, ascites fluid, Developmental Studies Hybridoma Bank). Cell death was additionally detected with TUNEL (Promega kit) for DNA fragmentation and 4’,6-diamidino-2-phenylindole (DAPI, 1 ug/ml) staining for nuclear fragmentation. All antibody labeling was done on deparaffinized slides; caspase and E-cadherin labeling was also done on whole mounts prior to wax-embedding, and E-cadherin on tissue fixed in NBF, processed in 30% sucrose in PBS at 4°C, and embedded, frozen and cryo-sectioned in OCT.

All antibody labeling was done using a standard PBT buffer (0.1 M phosphate buffered saline with 0.1% triton), a 30-minute pretreatment at RT in 3% hydrogen peroxide to quench endogenous peroxidase staining, a 5% horse serum-PBT blocker, mouse or rabbit primary antibodies, and a horse anti-mouse or goat anti-rabbit secondary antibody conjugated with peroxidase (1:200, Vector Labs), followed by diaminobenzidine (DAB) staining, and either hematoxylin or fast green counterstaining. BrdU antigen retrieval was done by immersing slides for 1 hour in 2 N HCl. Cleaved caspase-3 antigen retrieval was done by boiling slides for 5 minutes in a pH 4 sodium citrate buffer. Quadruple connective tissue and DAPI staining was done as previously described [50]. Plastic sections were embedded in Spurs epoxy resin, sectioned at 5 μm and stained with hematoxylin and eosin (H&E) by StageBio (formerly Histo-Scientific Research Labs). Whole mounts and dissected cartilages were stained with Alcian blue for cartilage and Alizarin red for bone using standard techniques. Sections were examined and photographed using a Leica DM 2500 fluorescent compound scope, and whole mounts with a Zeiss Stemi SV 11 dissecting scope.

Serial sections of wax embedded specimens were used as the primary data source because of the ease of capturing an entire anatomical region of interest and having reagents enter all parts of a cartilage. DAB staining was selected as the marker for all labels to allow its visualization alongside cell morphology, and to ensure longevity for repeated examination and use in future studies. Some specimens were labeled for caspase and E-cadherin as frozen sections or as whole mounts prior to being sectioned to confirm the staining observed in the wax-embedded sections, provide better section quality, and rule out wax sections failing to label as a result of the wax embedding process destroying their antigenicity. The thinner resin sections were included to provide cellular resolution not available from other methods.

### Quantification and description of cell features and labels

**Fig 1:**
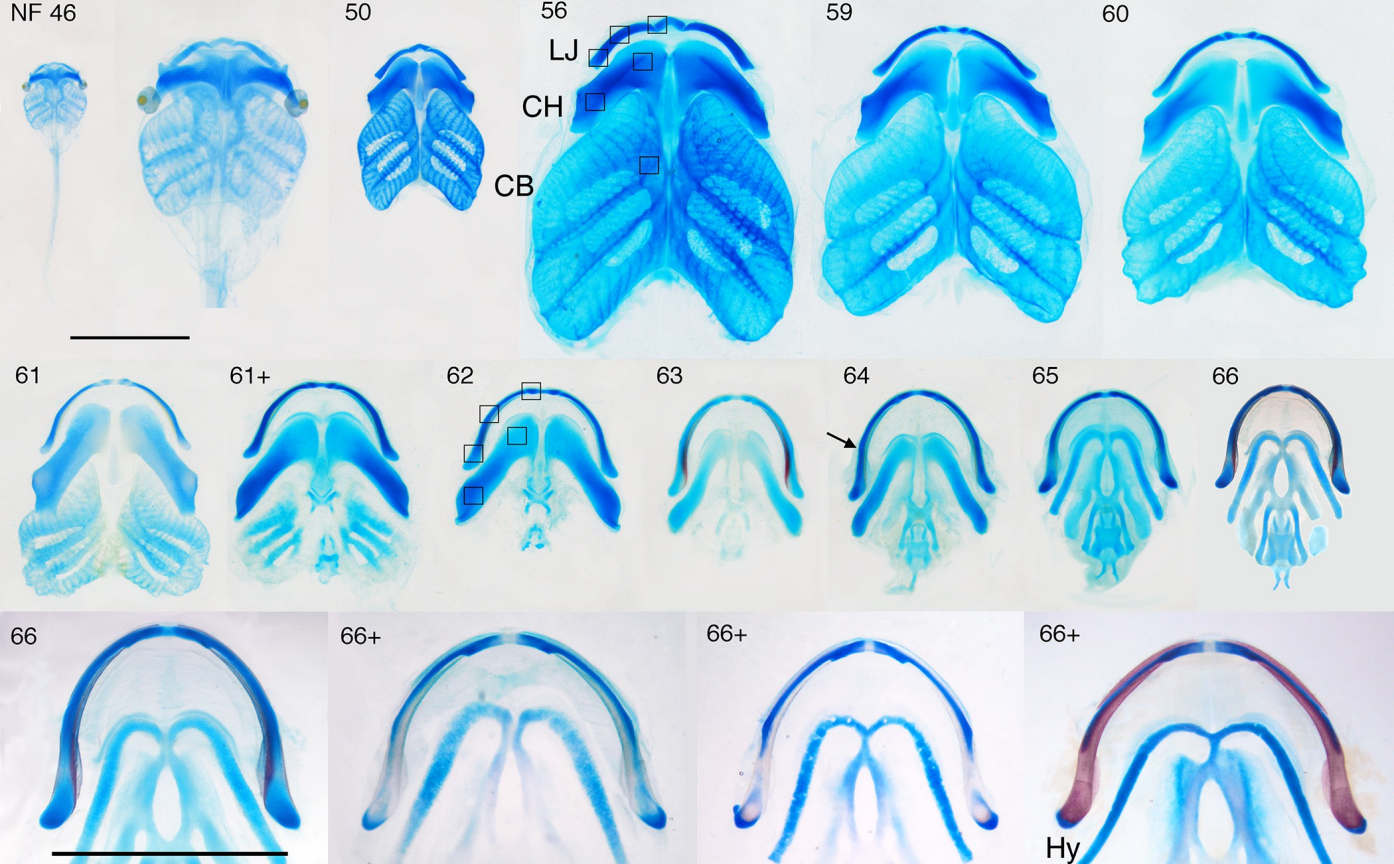
Ventral views of dissected, skeletally stained viscerocranial skeletons for NF 46–66+. Blue is cartilage, red is bone; LJ, lower jaw (the small, separate cartilage near the midline in NF 46–62 is the infrarostral, the larger, more lateral one is Meckel’s cartilage); CH, ceratohyal; CB, the first ceratobranchial (the four collectively are the branchial basket): HY; adult hyale; the boxes show the approximate regions selected in frontal sections for quantifying cell features; scale bars are 5 mm and apply to all panels except the second one in which the NF 46 skeleton is expanded to a comparable size with the NF 50 skeleton; arrow indicates the inflection point in lower jaw curvature; NF 46–59 covers tadpole growth, NF 59–66 covers metamorphosis, and the NF 66–66+ series spans approximately 5 days after NF 66. The NF 66–66+ series shows three postmetamorphic changes: loss of LJ cartilage just anterior to the joint, expansion of the adjacent dermal bones, and changes in CH histology. Modified with permission from [42].

Ontogenetic trajectories of BrdU label, cell size, shape and orientation, and percent matrix were quantified from frontal sections through six central regions of left and right cartilages: proximal, middle and distal regions of the lower jaw, proximal and distal regions of the ceratohyal, and the proximal region of the second ceratobranchial base (see boxes in Fig 1 NF 56 and 62 for approximate locations). The twelve regions were photographed under bright field microscopy at 10 X magnification and under phase at 20 X. The 10 X photos were used to count chondrocytes with BrdU-labeled and -unlabeled nuclei, and the 20 X photos were used to measure chondrocyte dimensions and orientations, and matrix accumulation using Image J; perichondral and adjacent cells were excluded from these data collections. To count BrdU-labeled cells, grid squares were superimposed onto each region, and the chondrocyte nuclei in each grid square were scored as labeled (DAB is brown under bright field microscopy and golden brown under phase) or unlabeled (hematoxylin is gray or blue). To measure chondrocyte dimensions through cell centers, the 30 largest chondrocytes in each 20 X photo were selected by eye, and the largest dimension of each and its orthogonal dimension were measured with Image J. The angle of each largest dimension was also measured relative to the long axis of the cartilage. Percent matrix was calculated using the area of each photo measured before and after the chondrocyte nuclei and cytoplasm had been erased with Adobe Photoshop. Left and right side data were combined for each region of cartilage. Five-nine specimens per stage were scored for BrdU label, and one specimen per stage were used for the other metrics; cell dimensions in the lower jaw were also measured in specimens sectioned at 5 μm to check for measurement artefact.

**Fig 2:**
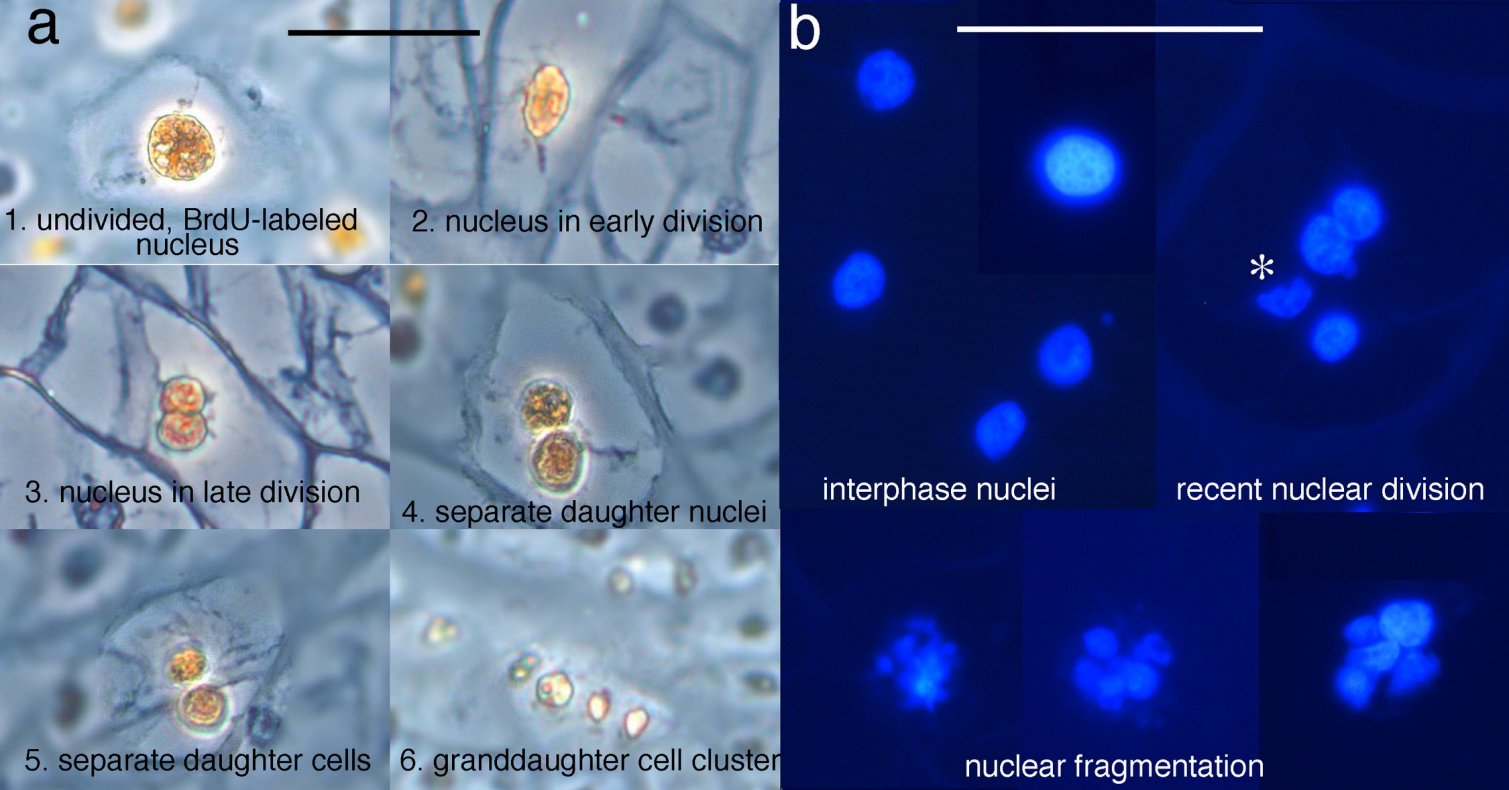
Criteria to score mitosis and nuclear fragmentation. (a) BrdU-pulsed chondrocytes at different stages of mitosis in phase micrographs. (b) DAPI-stained interphase nuclei, and nuclei showing signs of recent division and nuclear fragmentation in fluorescence micrographs. * indicates a nucleus that appears to have been fragmented by sectioning.

For BrdU-pulse treated specimens, the golden brown nuclei in 20 X phase micrographs covering the entire lower jaw and ceratohyal were scored for progression through the cell cycle using the following criteria: 1) an undivided nucleus, meaning an oval or spherical nucleus showing no sign of division, 2) an early nuclear division as indicated by a pear-shaped nucleus usually with one side smaller than the other, 3) a late nuclear division as indicated by a dumbbell-shaped nucleus, meaning two daughter nuclei that are distinct spheres but still connected, 4) two daughter nuclei that are no longer touching but the cell shows no sign of subdividing, i.e., it still has a cell membrane and surrounding matrix layer with the same shape, size and curvature as one with an undivided nucleus, 5) two separate daughter cells whose nuclei are separated by cell membranes with the same shape, size and curvature as those around an undivided nucleus, 6) a grand-daughter cell cluster, meaning 3–4 small, pale golden brown nuclei arranged in a round or oval cluster enclosed by matrix (Fig 2A).

The morphologies of chondrocyte nuclei were scored in photos of DAPI-stained frontal sections through a large portion of a lower jaw or ceratohyal. Nuclei were scored at 63 X for being in interphase, having undergone a recent division, and displaying karyorhexis or nuclear fragmentation, meaning that the nucleus has split into more than two fragments, or disintegrated into many, tiny fragments (Fig 2B). Nuclei that were faintly stained, small or appeared to be distorted or fragmented by sectioning were not scored.

All three cartilages and their adjacent tissues were examined visually in serial frontal and transverse sections from NF stage 44 to 67 for evidence of spatial patterns, e.g., a center, zone, gradient or directionality, in cell features or labels. Incubated BrdU label was examined in frontal sections for multiple specimens at each stage and in transverse plane for single specimens at NF 47, 53–4, 58–9, 62–3, 65, 66 and 66+; most other labels were examined for 1–3 specimens at each of representative early, mid, and late larval and mid metamorphic stages; TUNEL was applied to single specimens at NF 60 and 61. For non-BrdU treated specimens up to NF 62, the lower jaw and ceratohyal were sectioned and viewed frontally and the ceratobranchials transversely.

## Results

### Morphology and life history overview

Though the cartilage development and life history of *Xenopus laevis* are well described elsewhere [42, 47, 51], some background is required here to provide context for the cell-level descriptions of cartilage growth and shape change. Two days after hatching, the tadpole initiates a 5–8 week larval growth period (NF 46–59), during which the lower jaw, ceratohyal and branchial arch cartilages undergo gradual and subtle shape changes while increasing approximately 4–5 fold in linear dimensions (Fig 1). It then completes a 5–8 day period of metamorphosis (NF 59 to 66), during which all three cartilages undergo gradual and dramatic shape changes with no growth. The lower jaw becomes longer and thinner and changes from bow- to U-shaped, and the Meckel’s cartilage and infrarostral fuse. The ceratohyal shrinks in all dimensions and changes from a blocky to cylindrical shape. The branchial arch cartilage is lost to resorption. Whereas the first two cartilages are easily visualized in frontal and transverse sections, the branchial arch cartilage has a much more complex shape, a detailed explanation of which is provided in S1 Fig.

### Condensation, chondrogenesis and the emergence of perichondria and cell clusters

**Fig 3:**
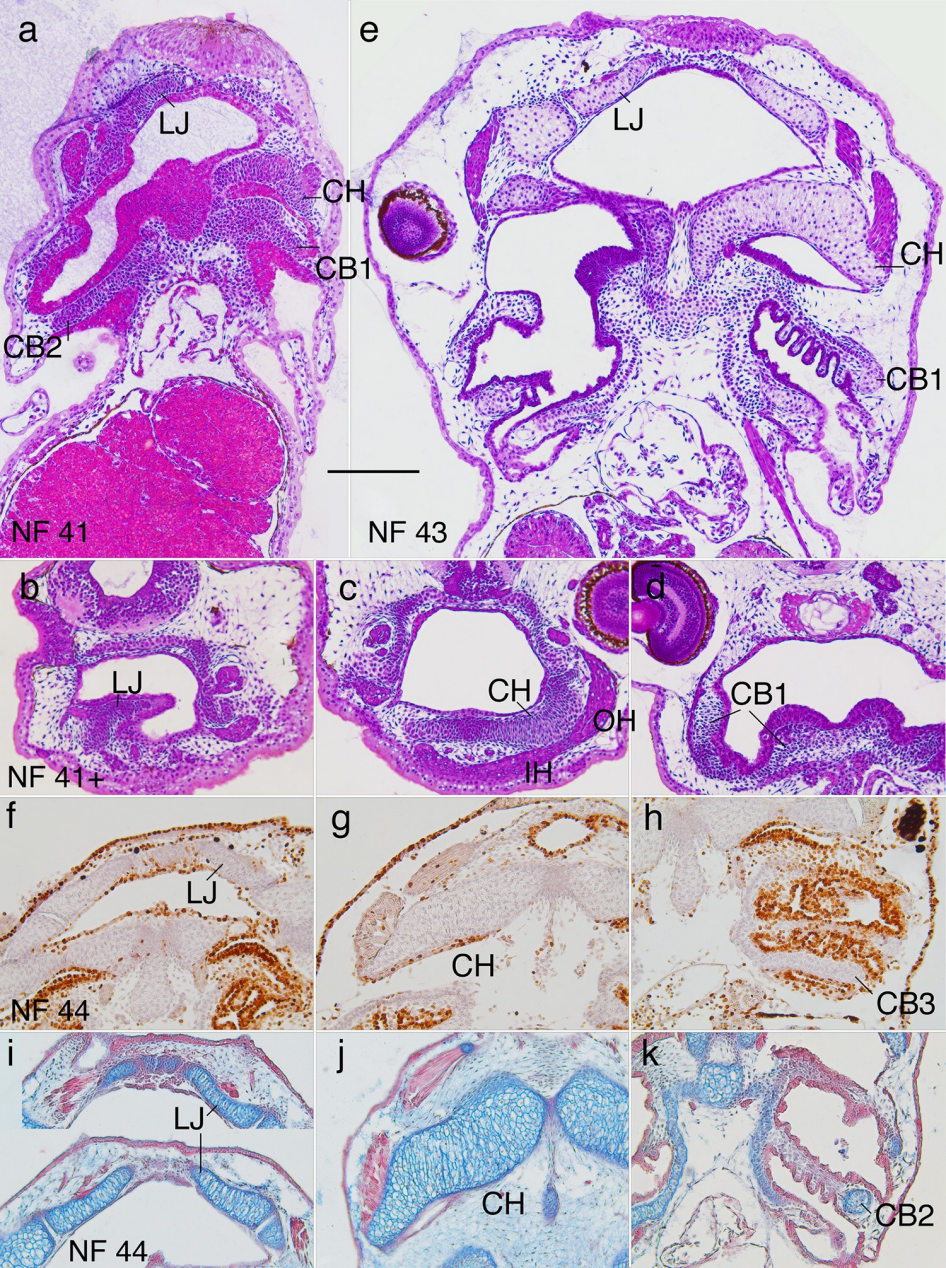
Morphogenesis of the lower jaw (LJ), ceratohyal (CH) and ceratobranchial (CB) cartilages from NF 41 to 44. (a and e) NF 41 and 43 frontal, resin-embedded, H&E stained sections. (b-d) NF 41+ transverse, resin-embedded, H&E stained sections. (f-h) NF 44 frontal sections with BrdU label, hematoxylin counterstain. (i-k) NF 44 frontal sections stained with alcian blue for cartilage and direct red for various collagens; IH, infrahyoideus muscle; OH, orbitohyoideus muscle; scale bar is 0.2 mm.

At NF 41 (~ 1 day posthatching or dph), the lower jaw, ceratohyal and ceratobranchial bases are present as cell condensations with recognizable larval shapes in the floor and walls of the pharynx (Fig 3A). The cells are polygonal, closely spaced, and mesenchymal, with little more than a nucleus and small periphery of cytoplasm; their long axes are transversely aligned in frontal view, meaning their greatest dimension is perpendicular to the long axis of the future cartilage. At NF 41+ (Fig 3B–3D), the ceratohyal cells are vertically elongated and aligned adjacent to the attachments of infrahyoideus and orbitohyoideus muscles [51, 52]. By NF 42 (~ 4 hrs after NF 41), cells below the surface begin to exhibit alcian blue-stained linings, spherical nuclei and conspicuously more cytoplasm. By NF 43 (~ 1.5 dph), all chondrocytes are larger, oval and transversely aligned in frontal view (Fig 3E). Also, all chondrocyte nuclei are spherical, similarly sized, and smaller than the flattened mesenchymal cell nuclei on cartilage surfaces. The alignment of the latter suggests the appearance of perichondria in all three cartilages.

The parts of the branchial basket dorsal to the ceratobranchial bases, meaning the walls and partitions, start to appear at NF 43 as condensations that are one or two cells thick (Fig 3E, 3H and 3K, see portions of partitions medial to CB1 and CB2). The condensations in the partitions are preceded by six ectodermal outpocketings that extend from the partitions into each flow chamber, and become filled with condensing cells (Fig 3E, 3H and 3K).

**Fig 4:**
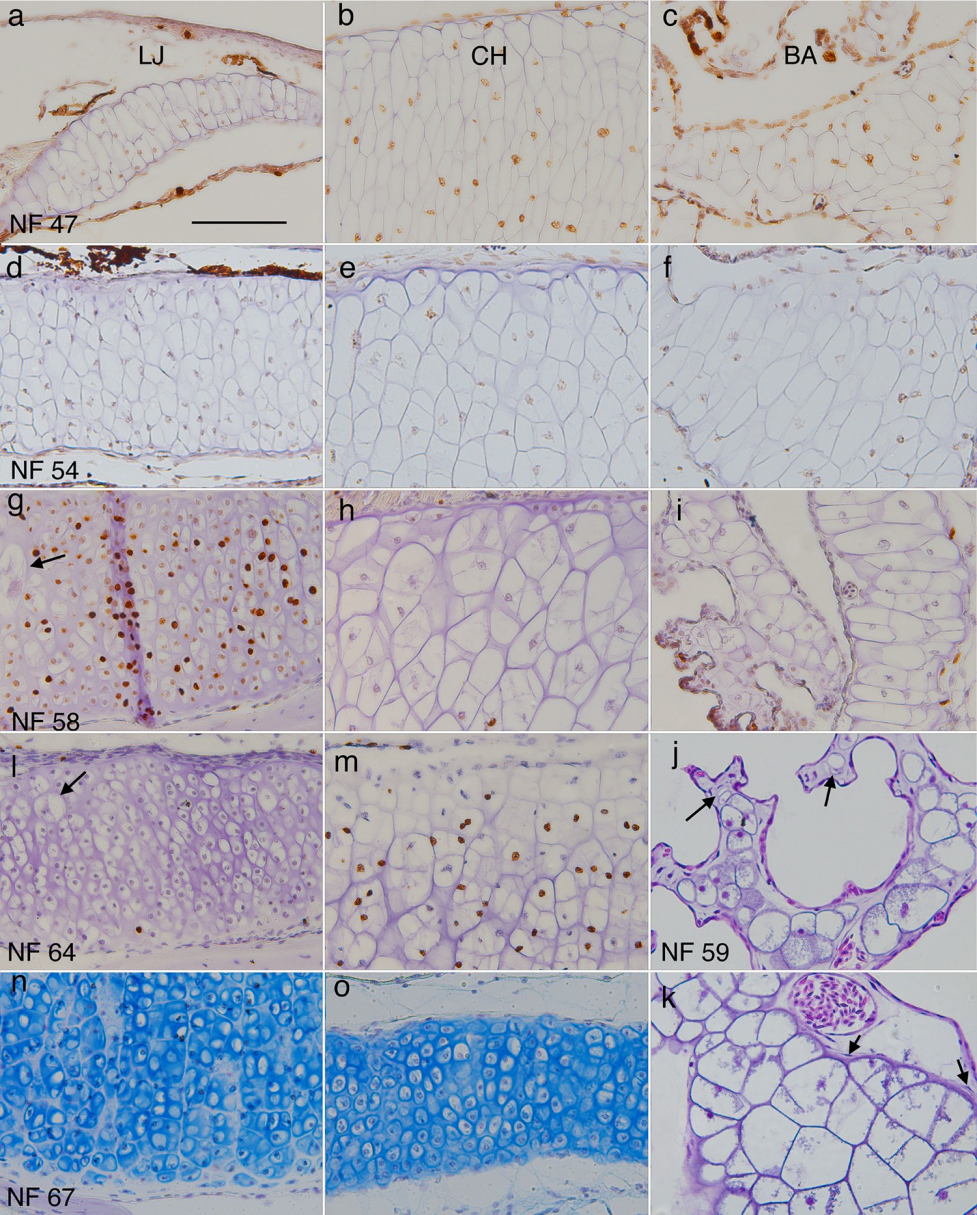
Representative sections of lower jaw (LJ), ceratohyal (CH) and branchial arch (BA) cartilages showing chondrocyte size, shape, arrangement, and matrix at different stages. (a-i, l-o) frontal, BrdU-labeled, hematoxylin or alcian blue-counterstained sections through middle portions of left LJ, CH and second ceratobranchial cartilages at NF 47, 54, 58, 64, and 67 (right is to the midline, up is anterior or lateral); arrows in g and l indicate large chondrocytes in the outer (more lateral) half of the cartilage. (j and k) transverse, resin-embedded, H&E-stained sections of branchial arch cartilages at NF 59. J shows an ornate process containing round chondrocytes of different sizes (arrows point to the smallest), capillaries, interstitial space, epithelia, and no perichondrium. K shows the transition from ceratobranchial base (lower right) to vertical rod (upper left), which is marked by a decrease in cell size, change from polygonal to more rounded cell outlines, and absence of perichondral cells (arrows) in the vertical rod. Scale bar is 0.1 mm.

The mesenchyme cells, chondrocytes and perichondral cells of all three cartilages are BrdU-negative from NF 41 to early NF 46 (Fig 3F–3H). By NF 44 (~ 4 hrs after NF 43, and 6 and 14 hrs before the onsets of feeding and air breathing respectively [48, 50]), the lower jaw and ceratohyal chondrocytes are conspicuously elongated and transversely aligned (Fig 3I–3K). Transversely aligned cell clusters start to become evident in frontal sections for the lower jaw, and in both frontal and transverse sections for the ceratohyal at NF 53–5, depending on the size of specimens (Fig 4D–4F). As the larval cell clusters are ellipsoidal in both planes of section, they are expected to have the three-dimensional shape of thin, stacked disks. The clusters remain evident in the lower jaw and ceratohyal until NF 64 and 65 respectively (Fig 4L and 4M), though their visibility varies considerably among specimens; cell clusters are never observable in the ceratobranchials.

### Quantification of cell features from NF 46 to 67

**Fig 5:**
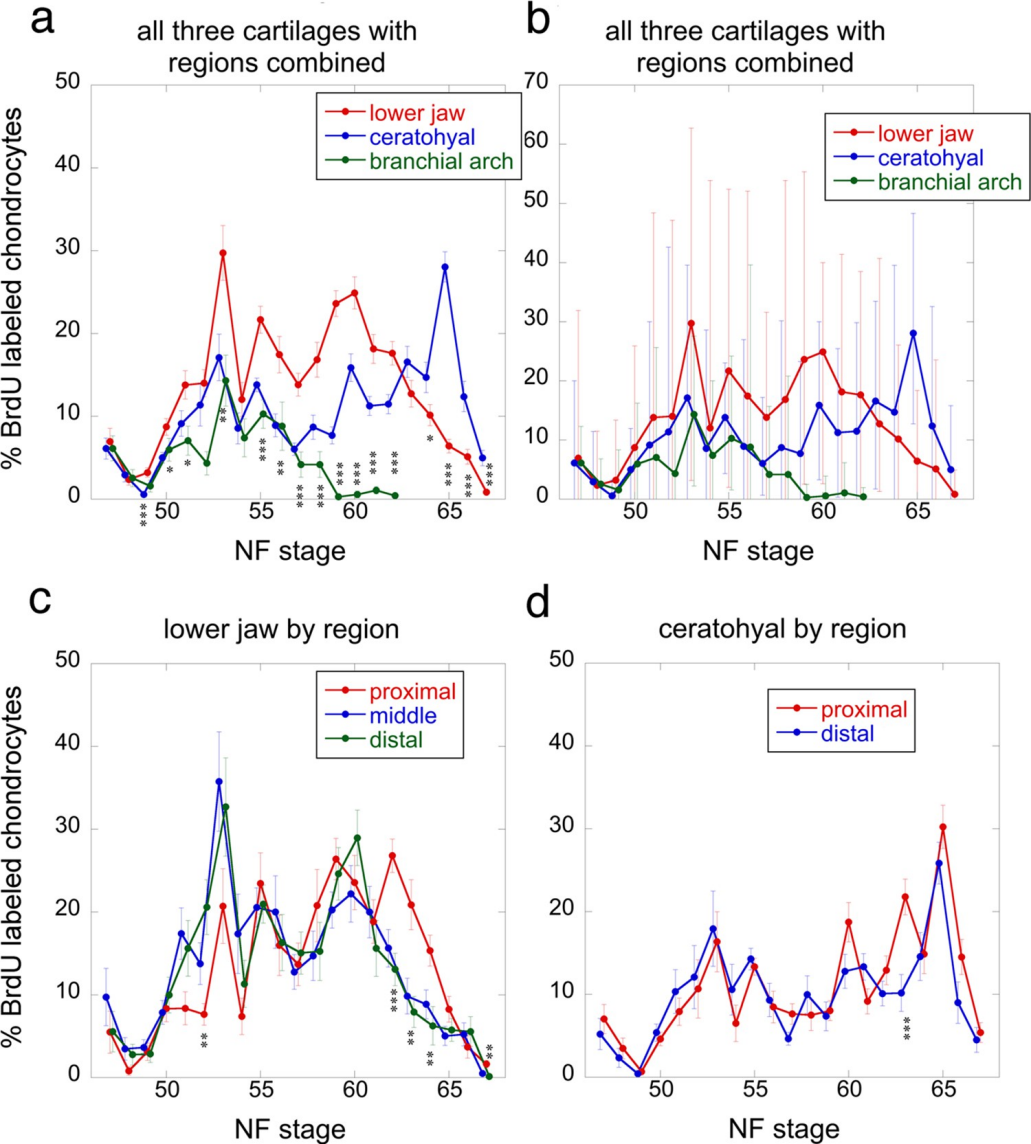
Percentages of BrdU-labeled chondrocytes for lower jaw, ceratohyal and branchial arch cartilages from NF 47 to 67. Each stage was scored from 5–9 specimens using 10 X photos of 10 μm-thick sections through central portions of left and right regions at the approximate locations of the boxes in Fig 1 NF 56 and 62, total number of specimens is 155; error bars show standard errors in a and c-d, and minimum and maximum values in b; asterisks indicate a significant difference among cartilages for a stage in a and among regions within a cartilage in c and d; one asterisk means p <0.05, two means p<0.01, and three means p<0.001 using ANOVA in R.

The regions used to quantify chondrocyte BrdU label, size, shape, and orientation, and matrix accumulation are shown in Fig 1 NF 56 for larval stages and NF 62 for later stages; representative sections are shown in Fig 4A–4I, 4L and 4M, though only the center portions of cartilages were used, and only large cells were quantified for size, shape and orientation. BrdU incubation treatments capture the percentage of chondrocytes that pass through the DNA replication (S) phase of the cell cycle in a 24-hr period (Fig 5 and S1 Table). The means of percent BrdU labeled chondrocytes for the three cartilages are significantly different at most stages from NF 49 onwards, with the lower jaw generally exhibiting the highest values and the branchial arch the lowest until NF 63, at which point the ceratohyal surpasses the lower jaw (Fig 5A). The trajectories for the three cartilages are similarly shaped in the tadpole growth period (NF 47–59), peaking at NF 53–54 and again at NF 55. The trajectories become more different at metamorphosis, with the lower jaw and ceratohyal peaking at NF 60, the branchial arch staying close to zero, and only the ceratohyal peaking at NF 65. The ranges between maximum and minimum values are large for all three cartilages at NF 47 and from NF 50 onwards (Fig 5B). Though some specimens at each stage have no or almost no BrdU labeled cells in all three cartilages, values for other specimens at the same stage commonly exceed 20%, and values for NF 52–59 lower jaws reach 50–60%. The regions within both the lower jaw and ceratohyal show significant differences at only a few stages (Fig 5C and 5D). Proximal regions tend to have lower values than distal regions until NF 55 and the opposite is true from NF 60–61 onwards.

**Fig 6:**
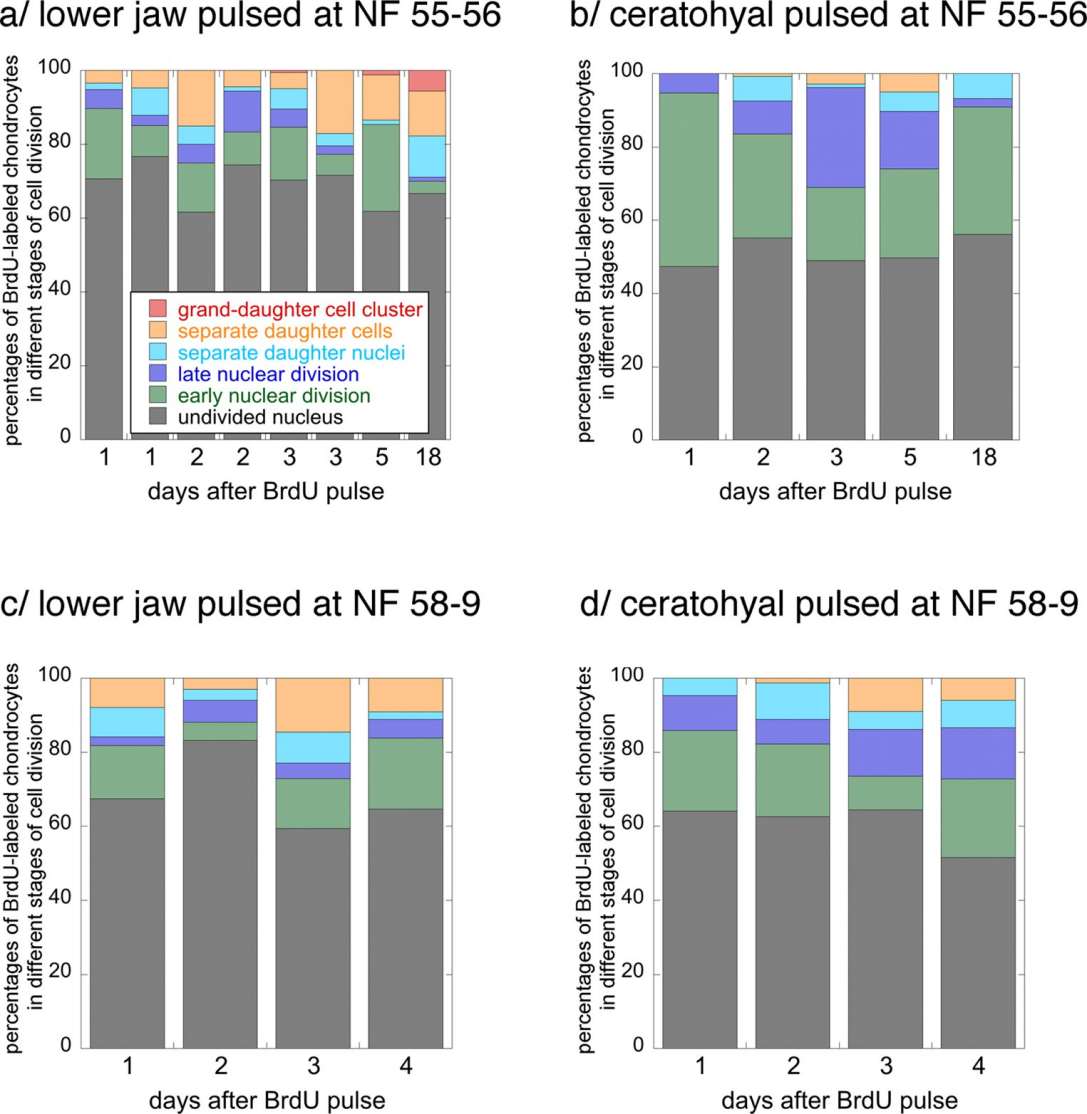
Percentages of lower jaw and ceratohyal chondrocytes progressing through mitosis in the lower jaw and ceratohyal at NF 55–6 and 58–9. Each column represents data collected from phase photos of cartilage from a single specimen following a 2-hour pulse of BrdU; see Materials and Methods and Fig 2A for descriptions and images of the six stages of cell division.

The BrdU pulse treatment labels chondrocytes that enter or are in the S phase of the cell cycle during the 2-hr duration of a pulse. By tracking this cohort after successive time intervals, one can learn how quickly chondrocytes progress through mitosis (Fig 6 and S2 Table). While the majority of lower jaw chondrocytes labeled at NF 55–6 do not progress to a stage of visible nuclear division within 18 days, about 12% do and another 6% have completed a second mitosis in that time. About 3–5% complete mitosis within one day, and 1% complete a second one within three to five days. Ceratohyal chondrocytes labeled at NF 55–6 take at least two days to complete their first mitosis and none appear to complete a second mitosis within 18 days, by which time the specimen has completed metamorphosis. Lower jaw chondrocytes labeled at NF 58–9 also progress a little faster than ceratohyal chondrocytes, though none in either cartilage complete a second mitosis within four days, by which time the specimen has reached NF 60. The lower jaw at both stages exhibits a higher proportion of labeled cells that appear not to enter mitosis than the ceratohyal.

**Fig 7:**
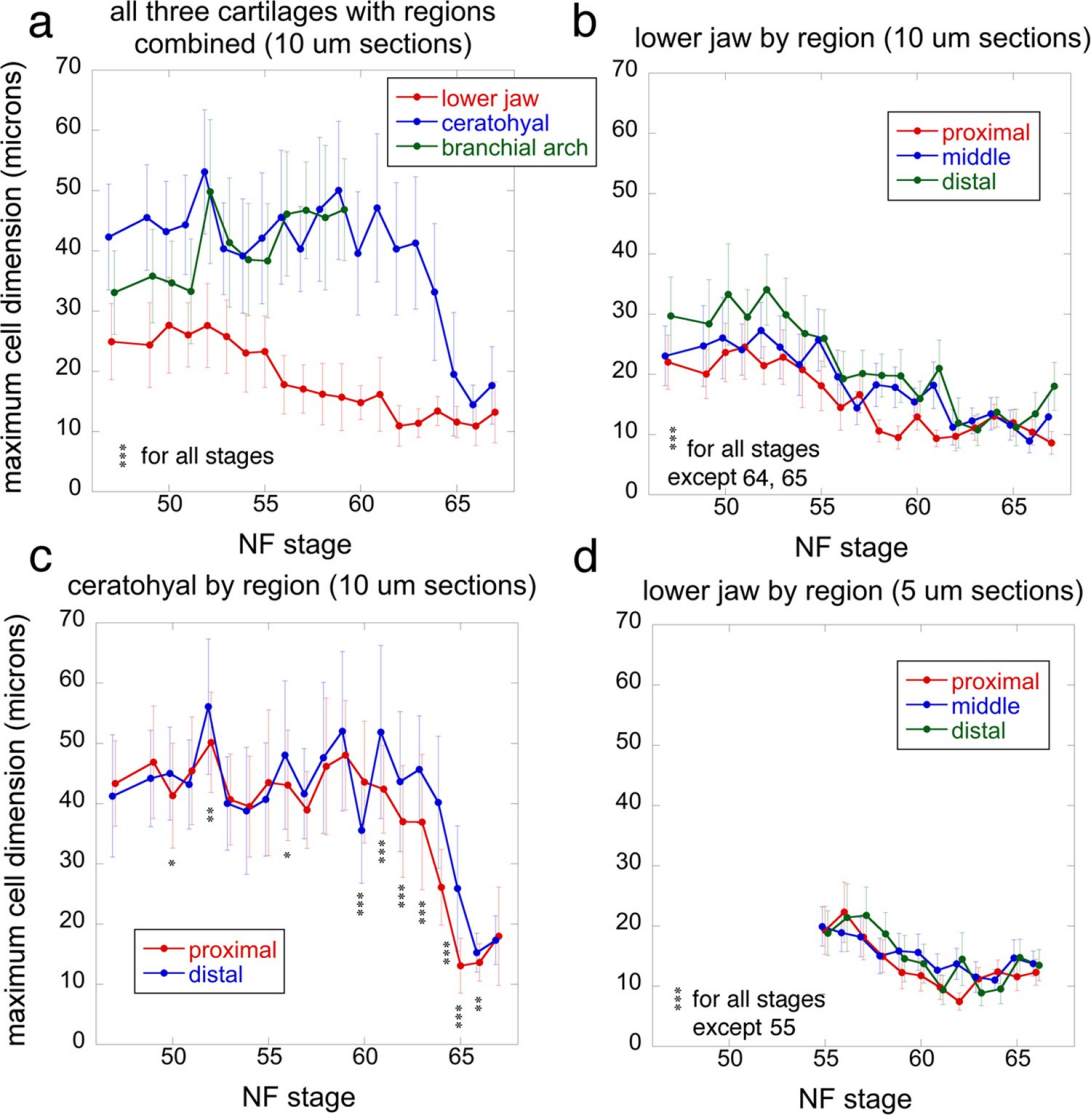
Maximum cell dimensions for lower jaw, ceratohyal and branchial arch cartilages at NF 47–67. Each stage was scored from 40 X photos of the left and right cartilages of a single specimen using the 30 largest chondrocytes in each photo; error bars show standard deviations as standard errors are generally too small to be visible; asterisks indicate a significant difference for a stage among cartilages in a and among regions within a cartilage in b-d; one asterisk means p <0.05, two means p<0.01, and three means p<0.001 using ANOVA in R.

The cell size trajectories are measured from photos used to count BrdU labeled cells; each stage is represented by left and right photos from a single specimen (Fig 7 and S3 Table). The average maximum cell dimensions for the three cartilages are significantly different at all stages (ANOVA, p<0.001), with the ceratohyal and branchial arch generally having the highest values and the lower jaw the lowest (Fig 7A). Lower jaw chondrocytes peak at 28 μm at NF 50–52, and then decrease slowly to 11–14 μm at NF 62, when they level off. In contrast, ceratohyal and ceratobranchial chondrocytes peak at 50–52 μm at NF 52, and stay above 39 μm until NF 59, the start of metamorphosis. At this point, ceratobranchial measurements were discontinued due to the onset of cartilage resorption and ceratohyal chondrocytes start to decrease rapidly, reaching 15 μm by the end of metamorphosis, NF 66. The lower jaw shows significant differences among regions at almost all stages in both 5 and 10 μm sections (Fig 7B and 7D). The ceratohyal shows significant differences among regions at a few larval stages and most metamorphic stages (Fig 7C). Proximal and distal regions have the lowest and highest values respectively at almost all stages for the lower jaw, and from NF 61 onwards for the ceratohyal. The cell size trajectories for lower jaws sectioned at 5 and 10 μm have generally similar shapes, magnitudes of values, and relationships among regions, though the regions differ less when sectioned at 5 μm (Fig 7B and 7D).

**Fig 8:**
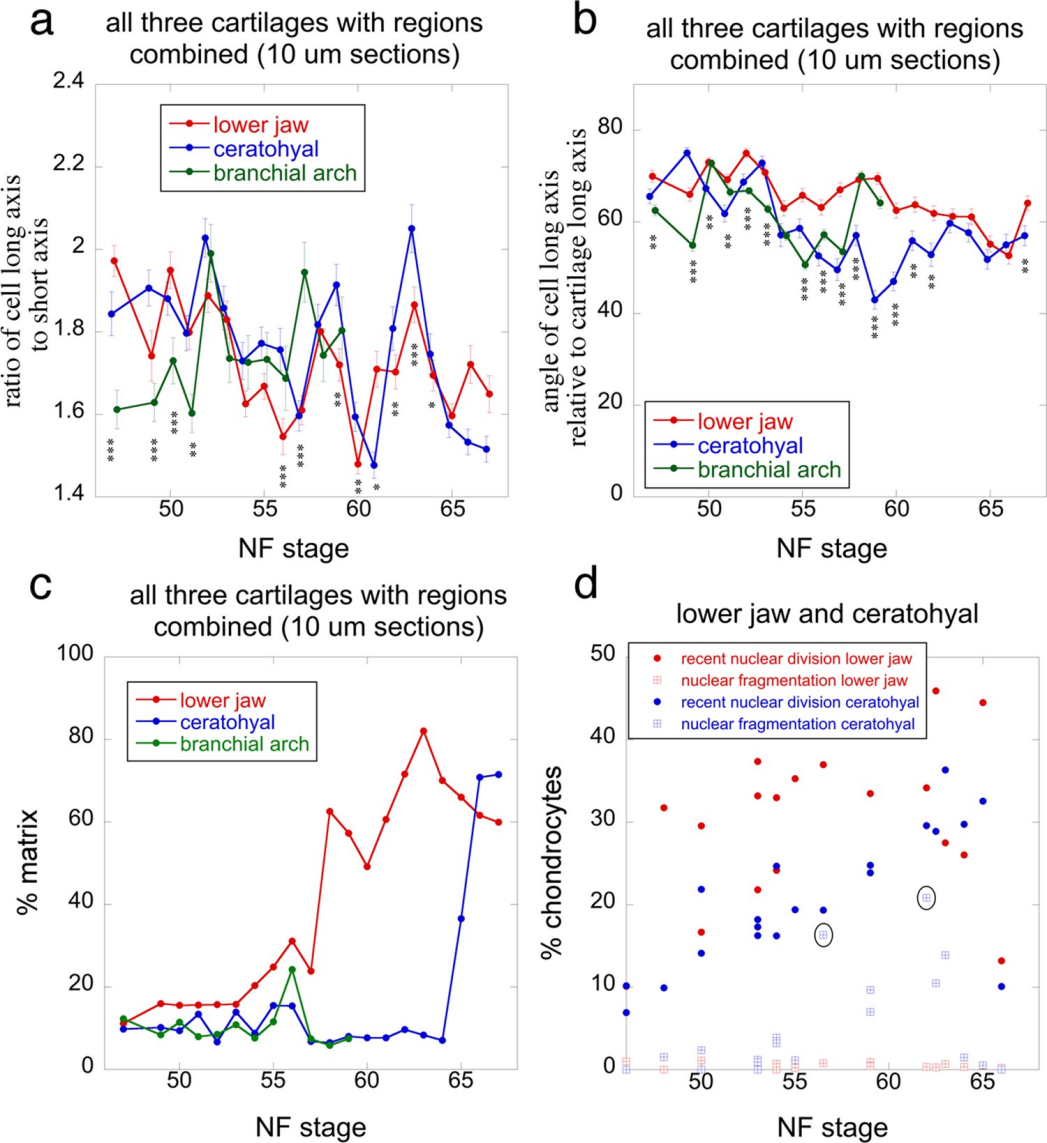
Cell shape, orientation, percent matrix and nuclear fragmentation for the three cartilages at NF 47–67. (a) ratio of cell long axis to short axis. (b) angle of cell long axis to cartilage long axis. (c) percent matrix. (d) percentage of chondrocytes showing signs of recent nuclear division and nuclear fragmentation. Data for a-c are from the same photos and cells used for Fig 7; error bars in a and b are standard errors; asterisks indicate a significant difference among cartilages for a stage; one asterisk means p <0.05, two means p<0.01, and three means p<0.001 using ANOVA in R. Data for d are from 63X DAPI fluorescence photos of a large central portion of each cartilage from 1–2 specimens at each stage; ovals indicate the two highest values; see Materials and Methods and Fig 2B for descriptions and images of nuclear fragmentation and recently divided nuclei.

The trajectories for cell shape, cell orientation, and percent matrix are derived from the same photos used to measure cell size (Fig 8A–8C and S3 Table). Cell shape is the ratio of a cell’s long axis to its short axis. Though the mean values are generally between 1.6 and 2, they fluctuate with stage for all three cartilages (Fig 8A). Whereas the lower jaw and ceratohyal mean values appear to decrease with stage and their fluctuations to increase in amplitude, the branchial arch values do the opposite. The mean angle of cell long axes relative to cartilage long axes is generally above 45° for all three cartilages. It is within 60-75° at early larval stages, and appears to decrease until metamorphosis when point it levels off. The percent matrix trajectories differ dramatically between the lower jaw and the other two cartilages (Fig 8C). The lower jaw trajectory is roughly opposite to its cell size trajectory (Fig 7A), with values starting low and increasingly steeply from NF 53 to 63. Ceratobranchial and ceratohyal values stay generally below 20% until NF 60 when ceratobranchial measurements were discontinued, and NF 64–66 when ceratohyal values increase rapidly. Percentages of chondrocytes showing DAPI-stained nuclear fragmentation differ dramatically between lower jaw and ceratohyal, staying ≤ 1% in the former and reaching 21% in the latter (Fig 8D). Ceratohyal values tend to increase with stage, peaking at NF 56.5 in the larval period, and NF 62 in metamorphosis. Percentages of chondrocytes showing evidence of a recent nuclear division are comparable to the percentages of BrdU-labeled cells following a 24-hour incubation (Figs 5A and 8C). Again, the lower jaw has higher values than the ceratohyal and ceratohyal values peak in late metamorphosis.

### General description of spatial patterns in cell features

**Fig 9:**
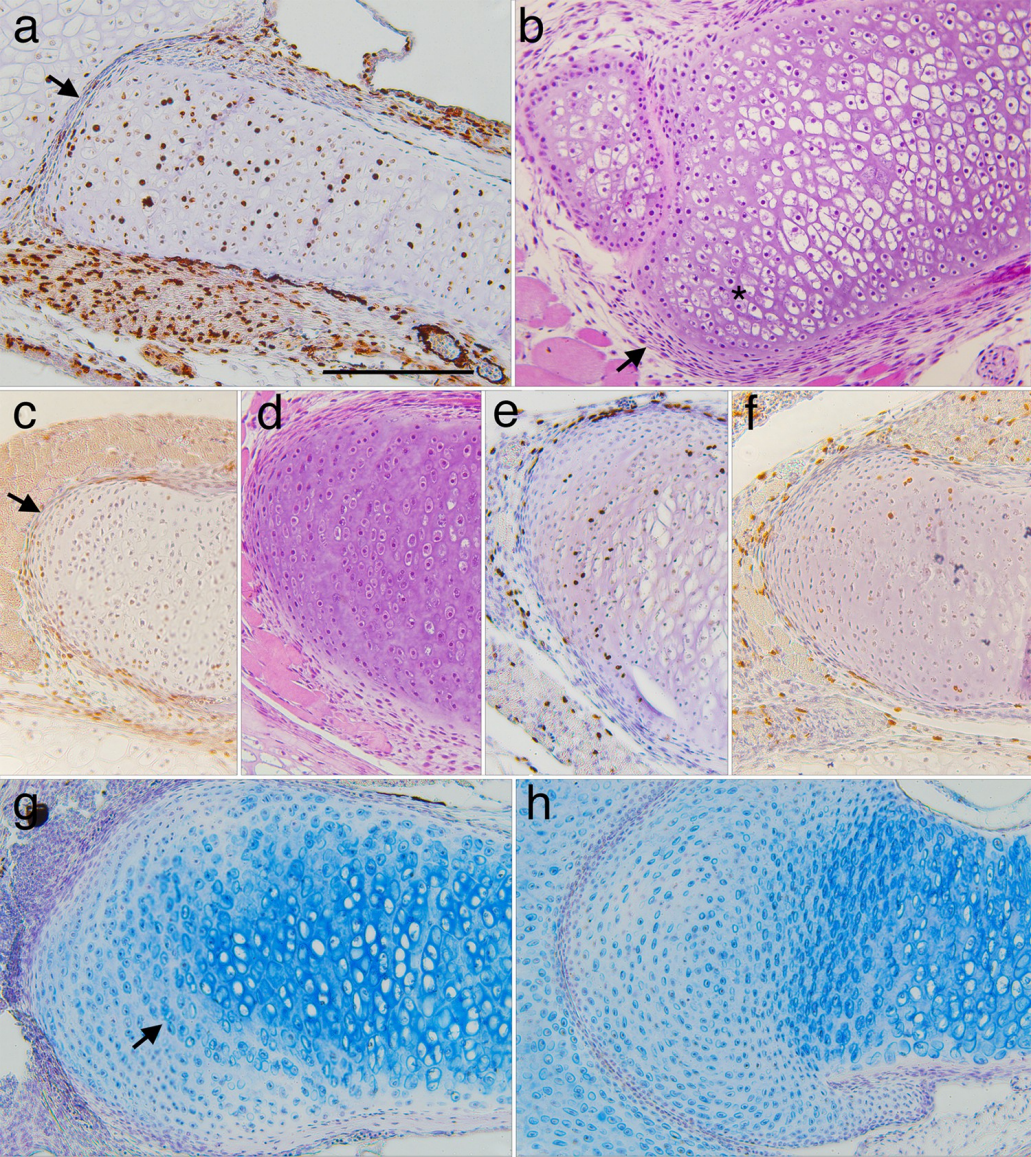
Frontal sections of the distal region of left lower jaws showing metamorphic changes in histology, shape and cell features. Right is to the front of the head and up is to the lateral side. (a) a late larval specimen (NF 58) showing BrdU labeling in transversely aligned cell clusters inside the cartilage, and the jaw joint with the palatoquadrate (arrow), which lies in the plane of the long axis of the cartilage at larval stages. (b) a resin embedded, H&E stained section at NF 59 (the smaller cartilage is the palatoquadrate) showing matrix and clusters of round chondrocytes near the distal end (*) and a layer of flattened cells outside the distal end (arrow). (c-f) a series from NF 61 to 64+ specimens showing the emergence of rows of small chondrocytes aligned with the distal end (arrow in c). BrdU labeling (c, e and f) remains low in perichondral cells, the newly emerging chondrocytes, and the larger, more proximally situated chondrocytes of the original larval cartilage. (g) a NF 67 specimen showing light Alcian blue staining of matrix around the outermost rows of new chondrocytes, strong staining around the larger, more proximally positioned chondrocytes, and moderate staining around the innermost row of new chondrocytes (arrow), which are larger and more circular than in outer rows. (h) the head of a humerus from the same specimen as g showing a similar pattern of chondrocytes and matrix staining. Scale bar is 0.2 mm.

**Fig 10:**
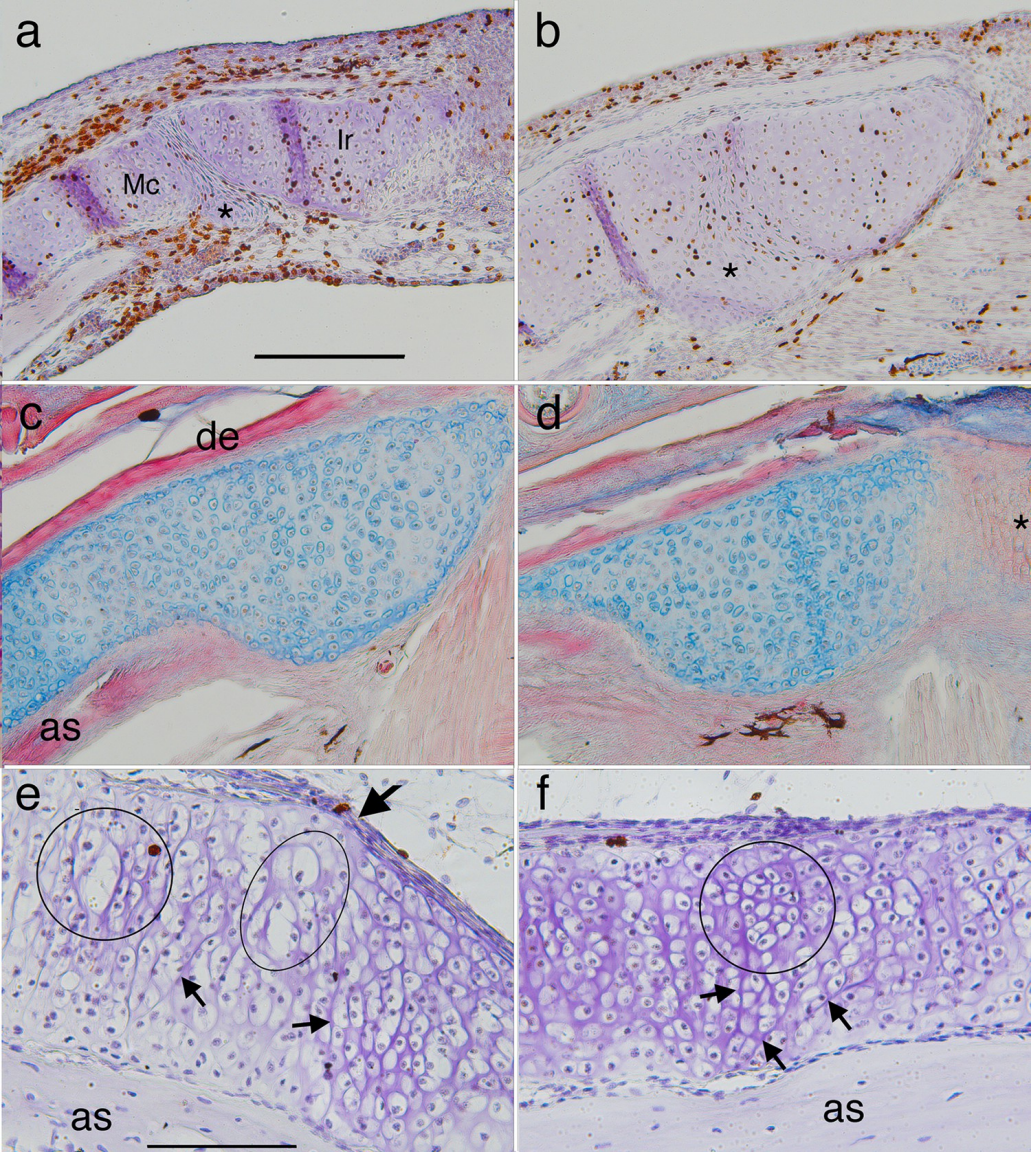
Frontal sections of the proximal (a-d) and middle (e-f) regions of left lower jaws showing metamorphic changes in Meckel’s cartilage (Mc) and infrarostral (Ir). Right is to the midline of the head in a-d and to the proximal region in e-f. (a) a late larval (NF 58) specimen showing a small condensation (*) posterior to the joint, and flattened cells in the joint space. (b) a NF 63 specimen showing the condensation having fused with both cartilages, and more matrix around cells in the joint space. (c and d) a NF 65/6 specimen showing alcian blue stained matrix, the dentary bone (de) on the outer edge, the angulosplenial bone (as) on the inner edge, and thin columns of small, unchondrified cells (*) in the midline. The absence of matrix in the midline is consistent with this part being bent repeatedly in opposite directions when feeding and ventilating. (E and f) the middle part of a NF 64 left lower jaw at two locations, the inflection point that has just emerged in its outer edge (e, large arrow here and in Fig 1 NF 64), and just proximal to this point (f). E shows large chondrocytes (circle and ellipse) in the outer (more lateral) portion of the cartilage and cluster boundaries running obliquely to its central axis in the inner portion (smaller arrows). F shows a round cell cluster (circle) in the outer portion and more obliquely aligned cluster boundaries in the inner portion (arrows). Scale bars for a-d and e-f are 0.2 and 0.1 mm respectively.

**Fig 11:**
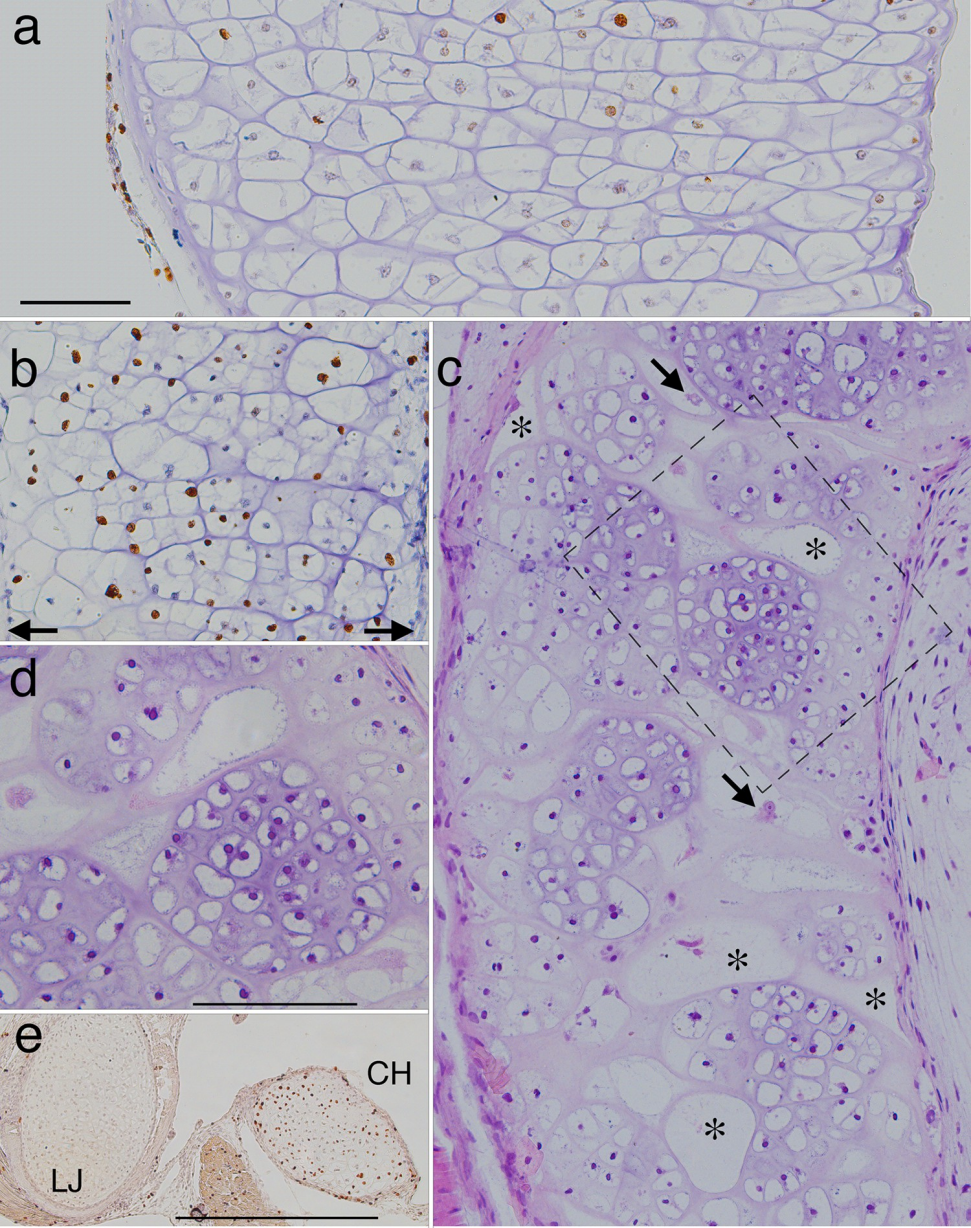
Sections through the proximal half of left ceratohyals showing metamorphic changes in cartilage histology, shape and cell features. Left is to the outside of the head and up is anterior in a-c and dorsal in e. (a and b) frontal, BrdU-labeled, hematoxylin-counterstained sections at late larval (NF 58) and late metamorphic (NF 64) stages respectively (arrows in b indicate the edges of the cartilage). (c) a frontal, resin-embedded, H&E stained section through the same region as a and b soon after metamorphosis (NF 66+). (d) a close up of the rectangular region outlined in c. (e) a transverse, BrdU-labeled, hematoxylin-counterstained section through the left lower jaw (LJ) and ceratohyal (CH) at NF 66. The transition from NF 58 to 64 (a to b) is marked by narrowing of the cartilage and most large chondrocytes acquiring more daughter nuclei and cells within their original perimeters. The transition from NF 64 to 66+ (b to c) is marked by the emergence of large, largely empty cell lacunae (*) that are interspersed across the width of the cartilage with discrete, equantly shaped cell clusters, and smaller lacunae that appear to contain cellular debris (arrows). While some lacunae resemble cluster outlines in b, others are transversely elongated with oval or more irregular shapes that generally conform to the curvature of adjacent cell clusters. The cell clusters contain many, small chondrocytes that have spherical nuclei and are separated by more matrix than the chondrocytes at NF 58. E shows high BrdU labeling in the ceratohyal, but not the lower jaw, at NF 66. Scale bars for a-d and e are 0.1 and 0.5 mm respectively.

**Fig 12:**
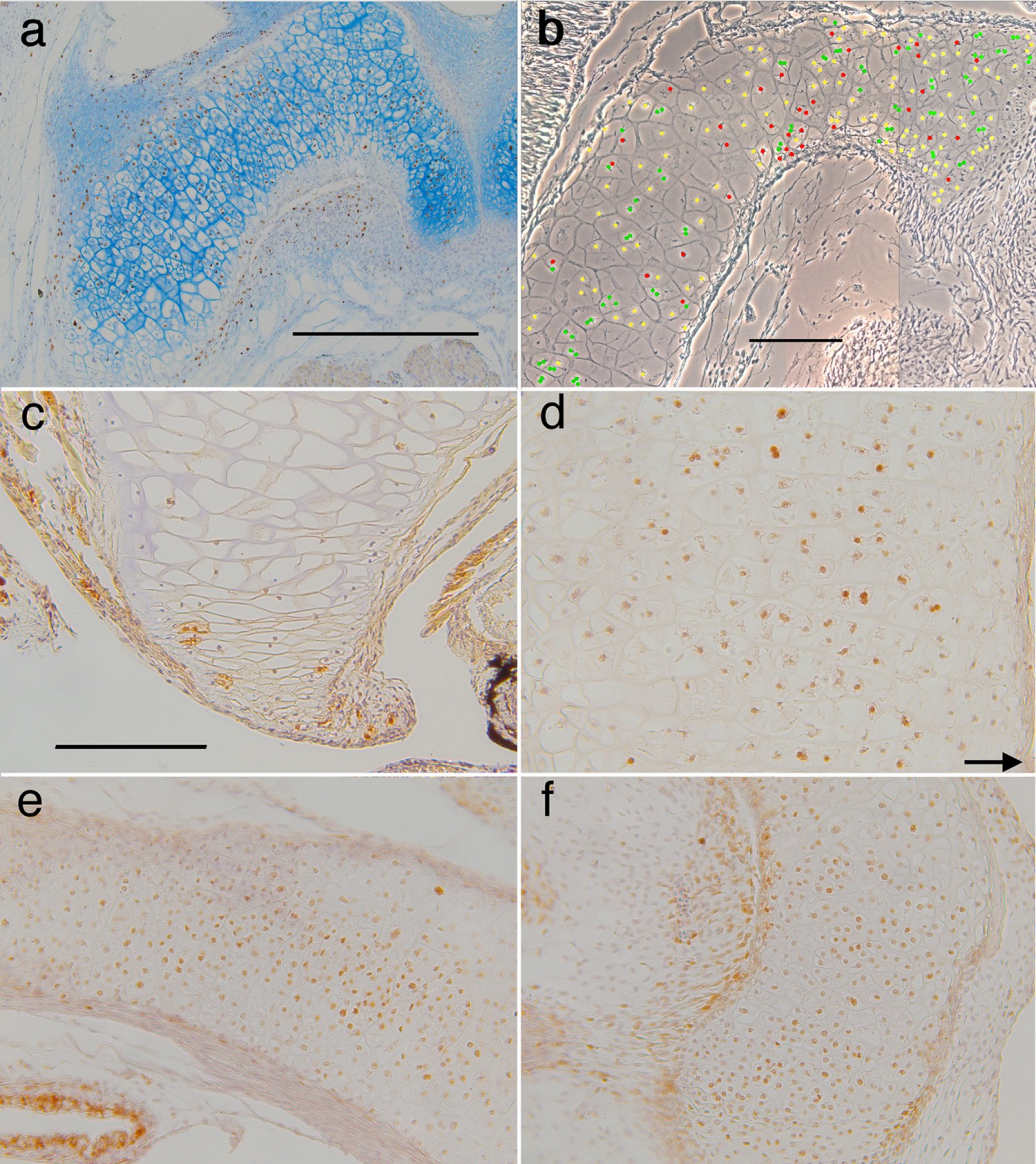
Frontal sections through left ceratohyals showing distributions of cell division and death labels. Anterior is up for a-d, and to the right for e-f. (a) BrdU labeled chondrocytes and alcian blue staining of matrix persist across the width of the cartilage to late metamorphosis (NF 65). (b) a phase micrograph at NF 63 showing the locations of DAPI-stained chondrocyte nuclei that appear to have recently divided (green), have undergone nuclear fragmentation (red), and be in interphase (yellow); see Fig 2B for scoring criteria. Recently divided nuclei are interspersed with ones exhibiting nuclear fragmentation. (c) capase labeling of a few peripheral chondrocytes in the distal tip at NF 63. (d-f) PCNA labeling of chondrocytes is strongest in the cartilage center at NF 62/3 (d) and 65 (e-f); arrow in d indicates the medial edge of the cartilage; scale bars for a, b and c-f are all 0.2 mm.

**Fig 13:**
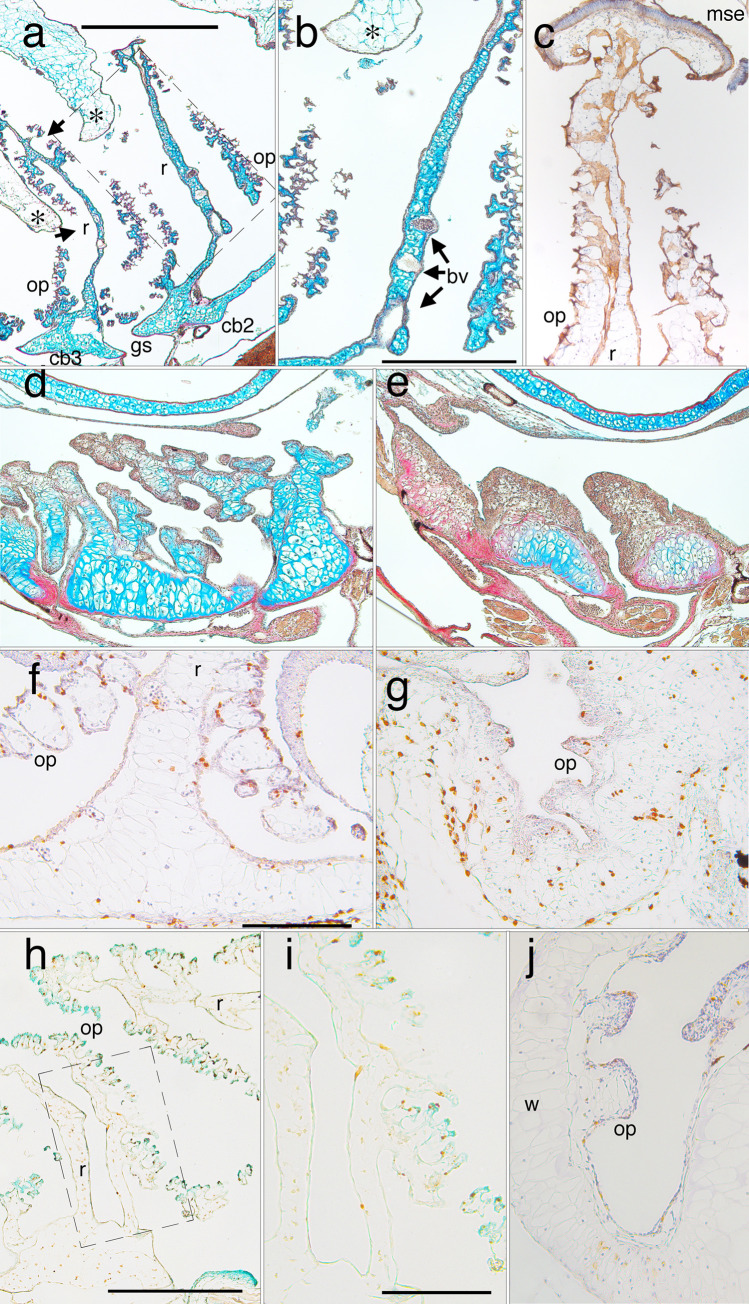
Transverse sections through left branchial arch skeletons showing metamorphic changes in cartilage histology, shape and cell features. Dorsal is up, medial is to the right. (a) Alcian-blue stained second and third partitions at a late larval stage (NF 58) showing ceratobranchial bases (cb2 and 3), the intervening gill slit (gs), and the vertical rods (r) that partition the space into flow chambers. The rods have anterior and posterior extensions that support filter surfaces comprised of ornate processes (op). Dorsal folds of pharyngeal epithelium (*) hang down into each flow chamber to force water to flow through the filter surfaces (arrows), into the spaces between ornate processes and rods, and out the gill slit. (b) a close-up of the rectangular region outlined in a, showing a rod, ornate processes and blood vessels (bv) within the rod. (c) brown E-cadherin staining of the squamous epithelium that lines rods and ornate processes at NF 59, and captures food particles. The columnar, mucous secreting epithelium (mse) that caps the inner walls and partitions transports the food particles to the esophagus. (d and e) progressive collapse of the dorsal portions of the partitions in early metamorphosis (NF 61 and 62), and the peripheral matrix in ceratobranchial bases ceasing to stain blue for chondroitin sulphate. (f) BrdU labeling of epithelial cells, chondrocytes, and perichondral cells in ventral portions at a late larval stage (NF 59). (g) BrdU-labeled chondrocytes at a mid-metamorphic stage (NF 62). (h and close up i) TUNEL labeling of epithelial cells, but not chondrocytes at an early metamorphic stage (NF 60). (j) caspase-3 labeling of epithelial cells, and less frequently and more faintly of chondrocytes at NF 62. Scale bars are 1 mm for a, 0.5 mm for b-e and h, and 0.2 mm for f-g and i-j.

The patterns for shape change in the lower jaw and ceratohyal will be explained in detail in later sections. Regarding cell division, aside from the subtle regional differences noted above for the lower jaw and ceratohyal, BrdU labeling reveals no visible zones or gradients of cell division in any of the three cartilages, their cell clusters or perichondria, and tissues adjacent to them during either growth or metamorphosis (Figs 4, 9–13). Perichondral cells appear to be less frequently labeled than chondrocytes, although the lower number of perichondral cells in sections and the difficulty of counting unlabeled ones precludes a quantitative comparison. PCNA labeling indicates a distinct spatial pattern in the ceratohyal at NF 62/3 and 65, showing stronger staining in centrally located chondrocytes (Fig 12D–12F).

Regarding cell size in larval growth, the ceratohyal at mid and late larval stages (NF 56–59) exhibits very small chondrocytes along its periphery, and its largest chondrocytes are in the center (Figs 4E, 4H and 11A, S3 Fig). The small chondrocytes are consistently BrdU negative and BrdU labeling of the largest chondrocytes in the center is not noticeably higher or lower than of adjacent cells. Although the walls and partitions of the branchial basket continue to grow in a ventral-to-dorsal direction, their surfaces never acquire perichondria, and remain defined by the thin matrix sheathes that line individual chondrocytes (Fig 4J and 4K, S2E and S2F Fig). All parts show dorsal-to-ventral gradients in cartilage thickness and chondrocyte size and shape. The ceratobranchial bases have large, polygonally shaped chondrocytes and more chondrocytes across their diameter than the rods (Fig 4K). The tips of walls, rods and ornate processes are a single cell thick and comprised of almost spherical cells arranged by size (Fig 4I and 4J, S2E, S2F Fig).

Regarding cell death, the lower jaw and ceratohyal are generally negative for caspase, though a very small number of chondrocytes (none in most sections) in the ceratohyal periphery are positive for active capase-3 at early metamorphic stages (Fig 12C). DAPI-labeled nuclear fragmentation in lower jaw chondrocytes is too infrequent at all stages to detect a pattern. Nuclear fragmentation in ceratohyal chondrocytes is observed across the width of the cartilage including at metamorphosis when the cartilage exhibits visible shrinking in whole mounts and the apparent loss of perichondrium and peripheral chondrocytes in histological sections (Figs 1 and 11A, 11B, 12A and 12B, S4 Table).

Connective tissue staining of the branchial basket shows that the four ceratobranchials are resorbed synchronously with each other in early metamorphosis and in a dorsal-to-ventral direction, starting with the ornate processes and dorsal edges of walls and partitions, and finishing with the more massive bases (Figs 1, 13A–13E). The branchial arch epithelium exhibits TUNEL and caspase labeling at NF 60 (Fig 13H and 13I), which marks the first visible signs of cartilage resorption. Branchial arch chondrocytes are TUNEL-negative at NF 60 and 61, but show a low frequency of caspase label from NF 60 (Fig 13J) until they become cellular debris at NF 65. BrdU label, which remains low in branchial arch epithelium and chondrocytes during growth, is noticeably higher just before their resorption (Fig 13F and 13G).

### Lower jaw shape change

In the lateral end of the lower jaw, new cartilage appears between the larval chondrocytes and perichondrium at NF 61–66 (Fig 9A–9G). The new cartilage can be distinguished histologically as small chondrocytes arranged in curving lines parallel to the perichondrium. Their appearance is not accompanied or preceded by elevated BrdU label in the perichondrium, though it is preceded by the accumulation of flattened, perichondral-like cells outside the perichondrium between NF 58 and NF 59 (Fig 9A and 9B). The new chondrocytes become separated by matrix before they start dividing, and the few incidences of BrdU label are not localized to the chondrocytes of any one line (Fig 9C–9F).

In the proximal end, the flattened cells in the joint between Meckel’s cartilage and infrarostral become surrounded by matrix, and both cartilages fuse with a small cartilage that appeared posterior to the joint between NF 58 and 59 (Fig 10A and 10B). BrdU label occurs throughout the region in early metamorphosis, and the infrarostral region at NF 66 is thicker, more bar-like and comprised of small chondrocytes that are separated by matrix and have no apparent alignment (Fig 10A–10D). The thickening produced by the fusion abuts the angulosplenial bone lateral to it (Figs 1, 10C and 10D). Though the left and right portions of the infrarostral are continuous at NF 56, by NF 65 the cells connecting them are small, aligned in long, thin columns, and do not stain with alcian blue (Fig 10D).

In the middle portion, the margins of cell clusters that were transversely aligned at NF 58 (Figs 4L and 9A) become more irregularly aligned by NF 64 and some are obliquely inclined both anteriorly and posteriorly (Fig 10E and 10F). Some chondrocytes in the outer (more lateral) portion of the cartilage are considerably larger than their neighbors at NF 58 and 64 (arrows in Fig 4G and 4L, outlines in Fig 10E). New, more equantly shaped cell clusters have appeared in the outer portion by NF 64 (circle in Fig 10F) near to the inflexion point in jaw curvature (arrows in Figs 1 and 10E). The new cluster flanked by oblique cluster margins (arrows in Fig 10F) suggests a wedge bending the cartilage. Chondrocyte division has ceased by NF 66 (Fig 11E), and both outer and inner portions of the cartilage appear comprised of almost square-shaped clusters of matrix-secreting cells by NF 67 (Fig 4N).

### Ceratohyal shape change

Whole mount and histological staining and caspase expression show the ceratohyal shrinking in all dimensions from NF 60 to 66, and this shrinking is accompanied by loss of the perichondrium, peripheral chondrocytes and matrix (Figs 1, 11A, 11B, 12A and 12B). Matrix staining becomes faint in the periphery, caspase is expressed in peripheral chondrocytes, and nuclear fragmentation is more common throughout the cartilage than at larval stages (Figs 8D, 12A–12C). Although BrdU label persists across the width of the cartilage until NF 65, PCNA label is noticeably stronger in more centrally positioned chondrocytes from NF 62/5 onwards (Fig 12A, 12D–12F). There is no sign of blood vessel invasion or large blood-borne cells appearing to resorb matrix at any stage.

Although the ceratohyal completes its shape change by NF 66, it continues to transform histologically after this stage (Figs 1 and 11). The changes in alcian blue staining between the first and fourth NF 66+ whole mounts (Fig 1) suggest that the adult hyale is not simply the retained central region of the larval ceratohyal. The visible gaps in stain in the third and fourth NF 66+ whole mounts correspond at a cellular level to lacunae that appear to be empty or to contain fragments of cellular debris (Fig 11C and 11D). Amidst the lacunae are newly emerging cell clusters that have polygonal shapes and straight borders with each other, appear randomly arranged along the length of the cartilage, and are more equantly shaped than the transversely elongated larval cell clusters (Figs 4H and 11A). The lacunae range in shape from round or oval in frontal and transverse planes to being highly irregular; some appear to be pinched by the growth of adjacent cell clusters and are bordered by a newly forming perichondrium. Their largest dimensions can exceed 100 μm and extend a third or more across the width of the cartilage. Some cell clusters contain cell clusters within them (Fig 11D, S2 Fig), and their component cells vary considerably in size, with some being as small as NF 43 chondrocytes, and others as large as NF 58 chondrocytes. Small chondrocytes are first observed in the central region at NF 62/3, along with the elevated PCNA label (Fig 12D), and BrdU label remains strong in this region for at least two days after NF 66 (Fig 11E). By one week after NF 66 (NF 67), the ceratohyal (or hyale, as it should now be called) has a uniform histology comprised of small round chondrocytes separated by matrix; there is no sign of empty lacunae interspersed with loosely coalesced clusters of variably sized cells (Fig 4O).

### T3-induced shape change in the NF 46 lower jaw

**Fig 14:**
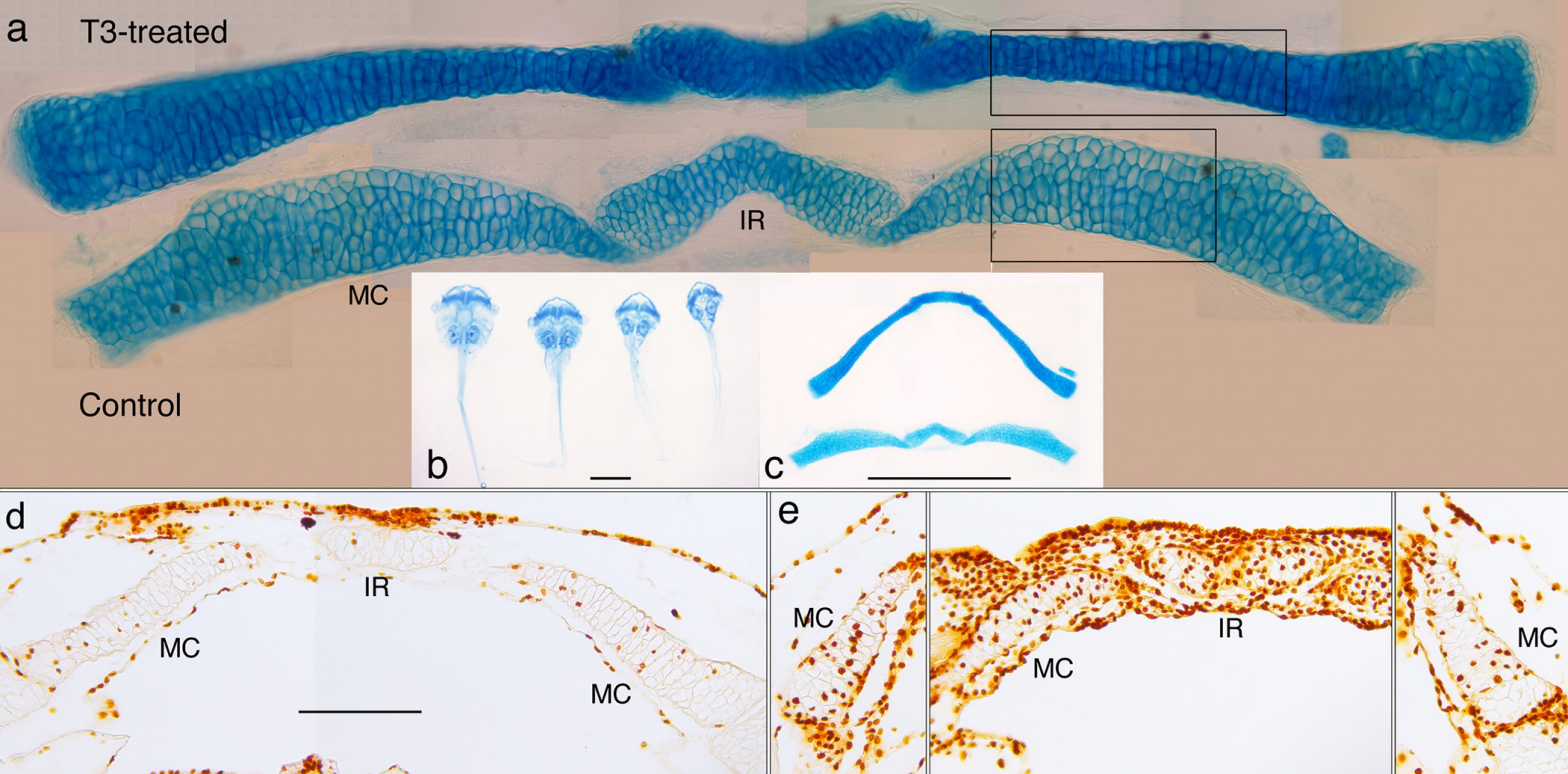
Effect of T3 on cartilage shape and cell behaviors in a NF 46 lower jaw. (a) the Meckel’s cartilage (MC) and infrarostral cartilage (IR) of a day 3, T3-treated specimen (upper) and a day 0, control specimen (lower) that were dissected from alcian blue-stained whole mounts of NF 46 specimens immersed in 50 nM T3. (b) ventral views of intact whole mounts treated for 1–4 days. (c) the cartilages after dissection. (d and e) frontal sections of BrdU-labeled lower jaws of day 3 control and day 3 T3-treated specimens respectively. Scale bars for b, c and d-e are 2, 1, and 0.5 mm respectively.

Since NF 46 tadpoles are commonly treated with T3 to study the lower jaw shape change, this treatment was repeated here to investigate the cellular basis of the shape change (Fig 14). Immersing NF 46 tadpoles at 10 days postfertilization in 50 nM T3 results in the lower jaw undergoing little increase in length or change in curvature, but thinning and thickening at different levels along its length to create a more uniform thickness overall. Comparing treated and untreated cartilages reveals that thinning in the middle region (boxes in Fig 14A) is the result of changes in cell size, shape and arrangement. Cells that are stacked as many as 6 across the width of the untreated cartilage are rearranged into stacks of 2 or 3 in the treated cartilage. Whereas the chondrocytes in the untreated cartilage are polygonal and their inner borders meet each other in a chevron pattern, the cells in the treated specimen are more cuboidal and their inner borders run parallel with the long axis of the cartilage. Though the T3-treated cartilage exhibits much more BrdU labeling throughout its length than the control (Fig 14D and 14E), only the labeled chondrocytes near to the infrarostral (left of the boxes) appear small enough to be products of an induced cell division. Cell division in this region is also consistent with its noticeable thickening. The T3-treated cartilage exhibits stronger alcian blue staining throughout its length, but not enough to noticeably change its thickness, based on the proximity of cell borders to each other and to the cartilage surface.

## Discussion

### Comments on techniques

Understanding the cellular basis of cartilage growth and shape change requires the collection and integration of data on all cell features that contribute to these processes [9, 10, 53, 54]. There is no technology currently available for following individual chondrocytes throughout larval growth and metamorphosis, let alone tracking their contributions to cartilage size and shape from dividing, growing, dying, secreting matrix, and forming cell clusters. Until such a technology becomes available, serial sections of wax embedded tissue remain the most time- and cost-effective means for collecting cell-level data throughout large, complexly shaped tissues over many stages and sizes. The challenge of detecting patterns in two dimensional views of complexly shaped cartilages can be offset by examining a large number of specimens (368 in this study) and limiting descriptions to replicated observations. The challenges of doing immunohistochemistry on wax-embedded tissues of a non-model organism can be offset by using multiple markers, fixation and processing techniques to corroborate findings and resolve between antibodies failing to bind antigens and antigenicity being lost in processing. One must also consider the usefulness of individual techniques and how to interpret their results in terms of cell behaviors that contribute to growth and shape change.

Whereas the BrdU label was consistently identifiable as positive or negative, the intensity of PCNA label in the lower jaw and ceratohyal varied to the point that it was often difficult to distinguish positive from negative. The high ranges in BrdU labeling found among specimens within a stage make sense in light of the inherently variable rates of amphibian larval growth and development especially in *Xenopus* [47]. Specimens with no labeled chondrocytes can be interpreted in two ways, all chondrocytes within a cartilage cycling in synchrony and the specimens being treated between S phases, or the chondrocytes not cycling together and the specimens being arrested in growth or metamorphosis. Not finding any cartilages or even cell clusters within cartilages with close to hundred percent label favors the second interpretation. The failure of many BrdU pulse-labeled chondrocytes to cycle through a mitosis within 4–5 days of undergoing DNA synthesis could also be a sign of chondrocytes cycling asynchronously. Cells whose DNA replication peaks during the 2 hours of BrdU exposure might incorporate enough BrdU to block or delay their cycling [55]. Others whose S phase overlaps with only the beginning or end of the 2 hours might receive enough BrdU to be labeled, but not impede their cycling.

Cell death occurs in a variety of ways, including apoptosis, autophagy and necrosis, and combinations of complementary but unrelated techniques are generally recommended to provide evidence for it [56–58]. To our knowledge, this study is the first to use TUNEL, caspase, and DAPI staining to detect natural cell death in *Xenopus* cartilages; resolving the exact pathway involved is not the intent of this study. The TUNEL results for branchial arch resorption, staining in cells adjacent to chondrocytes but not in chondrocytes, are consistent with findings on Meckel’s cartilage resorption in mouse embryos [59, 60]. Unfortunately, the autophagy markers thought to label chondrocyte cell death in those studies did not work here. Activated caspase-3 has been used to detect induced apoptosis in *Xenopus* branchial arch cells and others tissues [61]. Its natural expression in metamorphic stage branchial arch and ceratohyal cartilages here strongly suggests that most, if not all, chondrocytes in the former element and at least some in the latter enter apoptosis. The exceptionally low frequency of caspase expression in the ceratohyal is consistent with it being expressed early and briefly in the process of apoptosis [56, 58] and by cells that die asynchronously over a 5–8 day period. Nuclear fragmentation and other changes in nuclear morphology that can be visualized by TEM and fluorescing DNA dyes are widely used as evidence of natural cell death in numerous tissues [57, 62–64] including cartilage [65–68]. The nuclear fragmentation observed here, and TEM observations of degenerative changes and cellular debris inside lower jaw chondrocytes at the same stages [68] support the occurrence of chondrocyte cell death in larval growth and metamorphosis. Also, the early postmetamorphic appearance of large, largely empty lacunae in the ceratohyal has, to our knowledge, never been described before. We believe that their irregular shapes and arrangement, being much larger than the largest nucleated chondrocytes, being visible in well stained whole mounts, their occurrence only at one stage and in one cartilage, and some appearing to contain cellular debris and be deformed by adjacent cell clusters are all most parsimoniously explained as the lacunae being the remnants of greatly enlarged, dead or dying chondrocytes.

Chondrocyte size, shape and orientation and percent matrix are often measured in formalin-fixed sections using the cell perimeters that are defined by the most recently secreted matrix and that represent the maximum dimensions attained by cell growth, at least in the plane of section [34, 36, 54]. To our knowledge, the only way to measure the rate of matrix secretion at the level of individual chondrocytes is to label and quantify a specific matrix component produced over a set time interval [69]. However, in the absence of chondrocyte death and matrix destruction, percent matrix can be used to infer changes and differences in the contribution of matrix secretion to cartilage growth for a region of cartilage [54, 70]. The values calculated here are likely inflated to a small degree by the section thickness, with the degree of inflation depending on how many cell edges are captured tangentially, obliquely, and perpendicularly in a section. Having clarified how to interpret the different kinds of data, the next step is to integrate the results into programs of cell behaviors that explain the growth and shape change of each cartilage.

### The lower jaw

Based on the BrdU, cell size and percent matrix trajectories, growth of the lower jaw occurs primarily by cell division and cell growth, with little or no contribution from matrix secretion until midway through the larval period (NF 52), when cells start to decrease in size and secrete matrix. Since chondrocytes in viscerocranial and other cartilages typically increase in size with larval growth [71, 72], a steady decrease in cell size over many stages and much larval growth is most plausibly explained by successive mitoses producing daughter cells that consistently fail to grow to the size of their parent cells (Fig 4). The peaks in BrdU label at NF 52 and 55 (Fig 6A) suggest that larval growth involves two rounds of loosely synchronized mitoses, and the BrdU pulse results for NF 55/6 suggest that cell cycling might be as rapid as 2 days. The rise in percent matrix between NF 54 and 58 is consistent with cells switching from cell growth to matrix secretion in the later part of larval growth.

As the BrdU, cell size and percent matrix trajectories trajectories do not change noticeably at the start of metamorphosis, new cell behaviors must be initiated to explain the changes in cartilage length, thickness and curvature that occur in metamorphosis. This study finds evidence for chondrocytes being added to lengthen it at the lateral end and thicken it at the medial end. It also finds evidence for cell rearrangement in the larval middle portion occurring through the formation of new cell clusters.

Lengthening at the lateral end was predicted by classical morphologists [73] to accompany the backwards rotation of the jaw suspension cartilage and explain the shift from vertical to horizontal joint surfaces. The lengthening involves the appearance of lines of small chondrocytes that are separated by matrix and align with the inner surface of the perichondrium (Fig 9C–9G). These lines, which are first evident at NF 61 and increase in number to 10 or more by NF 66, are preceded by flattened cells aligning outside the perichondrium at NF 59. The entire perichondrium remains intact from early larval growth to the end of metamorphosis, and no part of it exhibits conspicuously higher BrdU label than adjacent chondrocytes and other tissues. If 10 lines of chondrocytes are added in eight days or less and if perichondral cells divide to produce them, one would expect the newest or outermost line at each stage to retain the BrdU label produced in the one day incubation period. This is not the case. At most two cells per line are BrdU-positive, and these are not necessarily in the outermost line (Fig 9E). There is also no sign of cells condensing and differentiating to form new cartilage outside the perichondrium. Collectively, these observations suggest that the perichondrium loses cells to successive waves of matrix secretion on its inner surface, and recruits undifferentiated cells on its outer surface. The perichondrium thus appears to move laterally like a wave from NF 59 to 66, transforming undifferentiated cells outside the cartilage into differentiated cells inside the cartilage. This contrasts with cell apposition in limb cartilage condensations and articular cartilage, where new chondrocytes are recruited from the perichondrium or cartilage surface, but not from outside the cartilage [9, 55, 74]. Whereas most Meckel’s cartilage chondrocytes derive embryonically from mandibular neural crest, the chondrocytes added to the lateral end have been suggested to derive from hyoid neural crest [75, 76].

Thickening of the medial end is also not unexpected given that this end includes the previously unchondrified connection between the infrarostral and Meckel’s cartilage, and is the one part of the adult lower jaw that is never buttressed posteriorly by bone [42]. The cellular changes described here suggest that the thickening involves chondrification of cells around the lateral and posterior edges of the infrarostral, fusion with the small posterior condensation, and continued cell division and matrix secretion inside the larval cartilages (Fig 10). The absence of newly aligned chondrocytes may indicate that cell apposition does not occur or that any cell alignment produced by it is obscured by rapid cell cycling.

This interpretation contradicts a study based on chondrocyte labeling that claims that new cartilage is added to lengthen the medial end [75]. If this were the case, the medial end of the larval lower jaw would have to be either resorbed or displaced laterally to allow for the addition of new cartilage. The current study finds no evidence of either. The infrarostral and its junction with Meckel’s cartilage remain distinguishable in whole mounts and sections throughout metamorphosis and do not shift laterally away from the midline (Figs 1 and 10). The previous study based their conclusion on nondetection in NF 63 whole mounts of a GFP marker for collagen II expression that was induced at NF 59 (73). The infrarostral becomes relatively smaller and the adjacent part of Meckel’s cartilage becomes relatively narrower as the animal approaches NF 59 (Fig 1). This is consistent with the medial end not growing and its cells expressing little collagen and thus little GFP at NF 59. This end also exhibits high cell division throughout metamorphosis and increasing cell size from NF 61 onwards, which is consistent with GFP being diluted in daughter cells. These factors, coupled with skin thickening, the deposition of additional matrix, and possible quenching of fluorescence from repeat viewing, draw into question the reliability of their marker.

In addition to terminal additions, the middle “larval” portion of the lower jaw appears to be lengthened and become more curved as a result of internal cell behaviors. This was suggested previously by the middle part of the cartilage bowing outwards when induced by T3 at stages just before natural metamorphosis [46]. The current study shows that the cell division and matrix secretion that drive larval growth continue as the cartilage begins to thin and tighten its curvature. Since the perichondrium stays intact and is increasingly flanked by growing dermal bones, the most plausible way for the larval lower jaw to continue cell division and matrix secretion while becoming thinner is by elongating along its central axis.

The remodeling induced here at an early larval stage provides evidence for the cartilage thinning involving cell rearrangement (Fig 14). Remodeling induced at NF 46 occurs with much fewer cells and less matrix than natural metamorphosis and before the formation of cell clusters. This makes it easy to visualize changes in cell shape and arrangement, but less likely that the resultant changes mimic the changes in natural development. Nonetheless, lower jaw chondrocytes appear specified to respond to TH by changing their shape and arrangement in a manner that supports cartilage thinning but not cartilage elongation (Fig 14). The naturally occurring changes described here provide evidence for a larger scale of cell rearrangement involving the formation of new cell clusters. Based on these observations, we propose that the tightening in lower jaw curvature is caused by new cell clusters that eventually fill the entire cartilage but appear first in the outer portion. This difference in timing causes the outer portion to lengthen relative to the inner portion, thus bending the cartilage. Understanding how the chondrocytes and matrix in the inner portion accommodate the bending, and how the replacement of larval transverse cell clusters by more equant adult ones might account for the overall thinning and lengthening calls for an imaging technology that can track the formation and fate of cell clusters.

The current results expand upon previous histological and TEM analyses at NF 57, 60, 63 and 66 [68] that also noted an overall decrease in cell size, increase in percent matrix, continuity of the perichondrium, transverse cell clusters being replaced by smaller, more equant ones, and small, densely arranged chondrocytes appearing in the lateral end. This research additionally found that the greatest increases in cell number and percent matrix occur at NF 60–63 and 63–66 respectively [70], and that the changes in cell size, number and percent matrix are TH-inducible [77]. The TEM analysis additionally found solitary, dying cells and cellular debris throughout the cartilage, cell columns that suggested a proliferative zone between the cartilage surface and center, and chondrocytes with single cilia interspersed with hypertrophic and dying ones in the cartilage center [68]. Since the cell division inferred to produce the proliferative zone was not confirmed by labeling, its exact occurrence and role in the shape changes remain unclear.

The current results help explain why NF 46 tadpoles treated with T3 show less jaw elongation than untreated controls [46]. T3 at this stage induces DNA synthesis but little, if any, mitosis in larval chondrocytes, and the cells become reshaped and more tightly stacked (Fig 14). There is also no sign of cell apposition being induced at the lateral end. These changes produce a more uniform width, but make the cartilage straighter, and do not increase its length. This finding reinforces the idea that the lengthening, thinning and tightening of curvature that occurs in natural metamorphosis cannot occur without the formation of new cell clusters inside the larval lower jaw and the recruitment of cells from outside its ends. Also, that the induced changes arise within three days and induced jaws fail to lengthen for seven days afterwards [46] would suggest that T3 or the cellular changes induced by it prevent the cell division and growth that would normally drive cartilage growth at this early larval stage.

### The ceratohyal and ceratobranchial

For larval growth, ceratohyal and ceratobranchial base chondrocytes undergo less cell division than lower jaw chondrocytes and use cell growth to attain a much larger size (≥ 50 μm) by NF 52, which they maintain with minimal matrix secretion until the start of metamorphosis. The small and large chondrocytes that arise respectively in the peripheral and central regions of the ceratohyal, and the gradients in cell size from ceratobranchial bases to ornate processes and dorsal edges might both be explained by unequal cell divisions and variable cell growth. As the large chondrocytes in the central ceratohyal are disk-shaped and stacked in three dimensions (S3 Fig), the small chondrocytes in the periphery are presumably needed to maintain smooth cartilage surfaces.

During metamorphosis, the ceratohyal resorbs in a surface-to-center direction and the branchial arch cartilage in a dorsal-to-ventral, fine-to-large-part direction. Unlike mammalian cartilages [74], the cartilages in this study lack blood vessels in the perichondrium, and the parts of the branchial basket outside of the bases lack perichondria. The directionality of resorption in both cartilages is consistent with TH entering chondrocytes to trigger cell death by diffusing across cell surfaces and perichondria from interstitial fluid. The dorsal-to-ventral, fine-to-large-part directionality of branchial arch resorption is consistent with the rods and ornate processes being lined with a well vascularized epithelium and perforated by numerous small blood vessels, and the entire capillary network being degraded during metamorphosis [78]. Branchial arch cell death could also be triggered indirectly, by fibroblasts near to the chondrocytes digesting matrix and disanchoring them as they do to notochord and muscle cells in tail resorption [79].

The emergence of the adult ceratohyal or hyale within the resorbing larval cartilage involves a subpopulation of chondrocytes that are indistinct histologically before metamorphosis, and appear to enlarge and form new cell clusters in late metamorphosis. Since the ceratohyal interior is never penetrated by blood vessels and the matrix remains intact well into metamorphosis, no cells appear to be introduced into the larval cartilage or moved centrally from its periphery during metamorphosis. The decrease in mean chondrocyte size that occurs over metamorphosis is most plausibly explained by large chondrocytes cycling rapidly with no cell growth or matrix secretion until the end of metamorphosis (compare Figs 4H and 4M, 11A and 11B). Using the ratio of average diameters for the largest cell at NF 58 to the smallest cell at NF 66 (S2C Fig) and assuming equally sized daughter cells and no cell growth or matrix secretion, the cells at NF 58 are estimated to undergo 6 or 7 mitoses by NF 66. Chondrocyte size reduction by rapid cell cycling is supported by the BrdU label increasing throughout metamorphosis and continuing for 1–2 days afterwards (Figs 5 and 10E).

The rapid cell size reduction in the ceratohyal appears to be combined with several features that do not occur in the lower jaw. Some peripherally positioned chondrocytes continue to undergo nuclear division and their surrounding matrix stains strongly for alcian blue until NF 65 despite being in a region that is soon lost to resorption. Dying chondrocytes throughout the cartilage leave behind largely empty lacunae that make the cartilage appear porous and at one point to be only ~ 60% living tissue. As the newly formed cell clusters grow by cell division and matrix secretion within their margins, they compress and occupy the spaces of the empty lacunae, ultimately fusing together within a newly forming perichondrium.

There is recent evidence that some epiphyseal plate chondrocytes do not necessarily die after they hypertrophy [80], and that human articular cartilage and skate fin cartilage have reserve and progenitor cells [81–83]. These findings support the notion that the large chondrocytes of the larval ceratohyal are predisposed or signaled to enter either cell death, or cell division and cluster formation in metamorphosis. Whether cell size, location and/or shape at the start of metamorphosis are involved in this decision requires knowing where exactly the parent cells of the cell clusters are located in the larval cartilage, what their phenotype is at that stage, and what triggers each metamorphic response. As with tail resorption, the disanchoring of chondrocytes by matrix resorption might play a role in activating those that die.

### General comments and comparisons with other vertebrates

The lower jaw, ceratohyal and ceratobranchial bases are all multiple cells thick when they condense, and they acquire perichondria and chondrify synchronously, though each cartilage exhibits minor regional differences in timing [84]. Their cells generally undergo similar changes as they transition from mesenchyme to chondroblasts and chondrocytes. The alignment of mesenchymal cells with newly formed muscle attachments in the ceratohyal (Fig 3C) is not evident at larval stages (S3 Fig), and could be related to the onset of muscle activity. The cartilages undergo little expansion as a result of the transitions in cell type (compare Fig 3A and 3I–3K), and cartilage growth by cell division and cell growth appears to commence primarily after the onset of tadpole feeding. That perichondral cells start to become evident right after condensation and before any appreciable cell growth, matrix secretion or cell division argues against their flattening as a result of physical forces being exerted from inside the cartilage [85]. The absence of perichondria in the fine parts of the branchial basket relates to the late acquisition of these structures both developmentally and evolutionarily (see below).

The cell behaviors inferred here to explain larval growth are not sufficiently resolved to explain the subtle shape changes that occur during growth, specifically the change in proportions and alignment of Meckel’s cartilage and infrarostral in the lower jaw, the emergence of concavities in ceratohyal and outer ceratobranchial surfaces [42], and the ongoing addition of vertical rods and ornate processes in the branchial basket. The decrease in relative size of the infrarostral and its gradual alignment with Meckel’s cartilage is likely facilitated by the medial region of the jaw having smaller cells, less matrix and less distinct cell clusters than the other regions.

The cell behaviors in *Xenopus* are generally consistent with what little is known about viscerocranial cartilage growth in other species. Early growth in recently hatched zebrafish [71] proceeds by cell division, cell growth and negligible cell death. This is preceded by a brief period of chondrocyte rearrangement that is not observed in *Xenopus*, and appears related to having finer, more intricately shaped condensations that permit some cell rearrangement after the onset of chondrification. Flatfish and mice both show transverse cell clusters in the early growth of their rod-like cartilages [72, 86]. Later differences in teleost fish relate to endochondral ossification. Growth proceeds by cell apposition, cell growth and matrix secretion until zones of chondrocyte hypertrophy and matrix resorption emerge, at which point the chondrocytes no longer grow or secrete matrix [37, 54].

Certain cell behaviors observed in amniote cranial cartilages are not observed here. Embryonic bird beaks are shaped by centers of cell division (growth zones) in epidermal and mesenchymal cells of the frontal nasal mass, and much of the variation in adult beak shape derives from differential control of these zones [16, 87–89]. Rod-shaped cartilages in mice embryos are elongated by cells intercalating from the perichondrium into transversely aligned cell clusters [86]. Though this behavior has not been ruled out for the *Xenopus* cartilages with perichondria, the continued division of chondrocytes within cell clusters, the increase in size of the clusters and chondrocytes relative to perichondral cells, the apparent separation of some clusters from the perichondrium, and two cartilages being in regular movement argue against it being a viable growth mechanism soon after embryogenesis.

Of the three programs proposed by Rose (2009, 2014) to account for cartilage shape changes in metamorphosing amphibians, this study finds no evidence of the first, cells condensing and chondrifying next to a larval cartilage, in the ceratohyal, and only a minor role for it in the lower jaw. This program likely accounts for formation of the paired alar and thyroid processes in the space previously occupied by the branchial basket (Fig 1) [42, 51], and for the additions of juvenile cartilage to the hyale in early postmetamorphosis [51] and elastic cartilage to the male larynx in sexual maturation [90]. The lower jaw and ceratohyal conform with the proposed second and third programs only to the extent that cell behaviors are more uniformly distributed in the former cartilage than the latter, and the perichondrium does not remain intact in the latter. The lower jaw exhibits more spatially localized behaviors than expected, including cell condensation and apposition at the ends and the earlier emergence of cell clusters on the outer side of the cartilage. The spatial patterning predicted for the ceratohyal, cell death peripherally and cell division centrally, is also incorrect as cell death and cell division occur in both regions.

### Significance for developmental biology and regenerative medicine

Shape regulation in the viscerocranial cartilages studied here requires that many cell behaviors be coordinated throughout a large population of similarly differentiated cells and over long periods of time, large increments of growth, and changes in use. This regulation appears to involve mechanisms that are intrinsic to cartilage and separate from those controlling its morphogenesis. The lower jaw and ceratobranchial are able to correct for shape abnormalities that are induced in morphogenesis during their subsequent growth and shape change [91, 92]. Bioelectric signaling involving ion channels in cell membranes is involved in their morphogenesis [93], as well as in size and shape regulation in zebrafish fin growth [94]. Also, cartilage is typically a viscoelastic material that is supported internally by matrix and fluid pressure and externally by a perichondrial sheath [95]. As they divide, grow, secrete matrix and die, chondrocytes are thought to generate and respond to physical forces that influence their behaviors on a cell-by-cell basis [69, 96, 97]. The temporal and spatial patterns described here provide a view into the cellular landscape where the role of bioelectric and biomechanical signaling might be investigated further at the levels of chondrocyte division, growth, and death, and their interplay to form and grow cell clusters.

Several new phenomena described here might also have broader implications for research on cartilage repair, regeneration and structure. These include cartilage growth in the absence of perichondria, cell clusters arising and coalescing to form a new cartilage in the center of a resorbing one, and chondrocytes regulating their cell size and their surface contacts to form bifurcated and tapered, single cell-wide structures. The latter process is further regulated across a large field of similar structures to grow and maintain the complex surfaces required for feeding and respiration. As these processes are likely to be tractable only in whole tissues, they are prime candidates for spatial transcriptomics and proteomics [98, 99].

Additionally, both the lower jaw and ceratohyal exhibit what we call chondrocyte rejuvenation, wherein chondrocytes that have grown to relatively large sizes (~ 28 and 53 μm respectively) undergo either slow cell cycling with matrix secretion or rapid cycling without matrix secretion to return to a prechondrogenic size (~ 10 μm). For the sake of comparison, limb growth plate chondrocytes are typically 10–30 μm in greatest dimension [34, 36, 100]. The only other chondrocytes known to reach 50–60 μm are in the growth plates of exceptionally long-limbed vertebrates like bats and jerboas [39, 40]. Mammalian cartilage generally does not regenerate, and the self-maintenance of articular cartilage is the subject of great biomedical concern [69, 81]. Understanding the genetic regulation of chondrocyte rejuvenation in *Xenopus* might help to develop cartilage repair treatments. Degenerative osteoarthritis involves abnormal chondrocyte proliferation leading to clonal cell clusters that expand at the expense of adjacent matrix [69]. Understanding the rapid emergence of the ceratohyal cell clusters might similarly inform osteoarthritis treatment and prevention.

### Significance for functional and evolutionary morphology

Understanding how cell behaviors accomplish cartilage growth and metamorphic remodeling is key to understanding the evolutionary origin and diversification of amphibian metamorphosis, and the evolvability of cartilage shape [11, 12, 101]. *Xenopus* stands out in two respects: 1) the unexpected superimposition of cell behaviors for shape change onto ones for growth, and 2) the unprecedented exploitation of chondrocyte cell size to diversify cartilage shape, structure and histology.

The superimposition of cell behaviors is evident in two ways. Peripheral chondrocytes in the ceratohyal complete two or more nuclear divisions in the 24 hours just before the region is lost to resorption (Fig 12A), and branchial arch chondrocytes show higher BrdU labeling during arch resorption than at any larval growth stage (Fig 13F and 13G). This superimposition contrasts dramatically with the radical metamorphoses of insects, sea urchins, and nemertean worms, wherein larval tissues are lost quickly to whole-scale cell death while new parts arise from cell proliferation in “set aside” adult rudiments [102–104]. The invertebrate metamorphoses exhibit perhaps the greatest duality allowed in evolution, i.e. two very different animals in one, and a quick and efficient means of exercising it, as the larval anatomies and body plans are small and simple, and provide little, if any, constraint upon the more complicated adult ones. Vertebrates in general are bound to much greater cellular continuity throughout life by building large, complex bodies through prolonged phases of cell migration, cell signaling, and tissue morphogenesis. Evolving a metamorphosis requires a choice between replacing specialized larval cartilages with new adult ones, or retaining them and transforming their size, shape and histology to meet adult needs. Whereas frogs adopted the first option for the ceratobranchial, and the second for the lower jaw and ceratohyal, salamanders evolved both options for the ceratobranchials [44]. However, aside from the rather spectacular examples of ceratobranchial replacement in plethodontids [66] and hyoid arch reshaping in hynobiids and dicamptodontids, salamanders do not generally exhibit the same degree of shape change in their first and second arch cartilages as frogs and their third arch remodeling involves largely resorption [12, 45, 101].

The programs described here present a starting place for investigating differences in the evolvability of cartilage growth and shape change across vertebrates. This starting point, however, is a rather atypical amphibian with unusual functional needs [11, 12]. As a filter feeding, gill-less tadpole with an exceptionally large head, *Xenopus* moves its jaw and ceratohyal almost continually to pump water through a relatively large tadpole mouth and very large branchial complex that functions in both food collection and gas exchange [105–107]. As an aquatic, tongue-less adult, *Xenopus* uses its hands to push food into its relatively small frog mouth, and its throat skeleton to breath air as other frogs do, but not to protrude and retract a tongue. The lower jaw program appears to use moderate chondrocyte growth and slow rejuvenation with matrix secretion to increase mechanical strength throughout growth and metamorphosis, and allow for a quick, moderate shape change that does not impede function. The ceratohyal program uses exceptional chondrocyte growth and rapid rejuvenation to produce a massive, but light larval element that can move a large volume of water efficiently, and be drastically transformed in shape, histology, and size. The branchial arch program exploits the ability of cartilage to grow at single-cell dimensions to evolve an exceptionally large, complex, and delicate filtering skeleton that compensates for the loss of gill filaments [12, 108, 109].

That the cell behavior programs exhibited by *Xenopus* can be exploited for much greater ontogenetic divergence and for significant phylogenetic diversification is evidenced by the wide array of larval and adult shapes that have evolved in frog viscerocranial skeletons [12, 45]. Some frogs have tiny lower jaws as tadpoles and huge ones as adults, others lose the ceratohyal at metamorphosis, and some have very simple branchial arch skeletons. This high diversity could be tied to cell behaviors for shape change being superimposed onto ones for growth, and to chondrocytes undergoing dramatic reductions in size, as these factors allow for significant shape change without the functional and energetic costs of cartilage replacement. Whether salamanders, lampreys and other metamorphosing vertebrates evolved the same flexibility in their cartilage remodeling programs remains to be seen. Unravelling the rules and limits that the repertoire of cell behaviors available to chondrocytes imposes on skeletal evolution awaits more comparative study of how they grow and remake cartilages.

## Conclusions

This study is the first attempt to describe and quantify the cell features that underlie cartilage growth and shape change from early postembryogenesis to a juvenile stage for any vertebrate. The results are interpreted in terms of specific cell behaviors, which are integrated into programs to explain the larval growth and metamorphic shape change of three functionally important cranial cartilages. The results provide a conceptual framework and starting point for phylogenetic comparisons, and a baseline for investigating thyroid hormone-induced remodeling, and intrinsic shape regulating mechanisms involving cell communication, biomechanics and bioelectricity. They also reveal four phenomena not previously described in vertebrates: chondrocytes being rejuvenated after a lengthy growth period by dividing back to their prechondrogenic size and shape, a cranial cartilage that lacks a perichondrium and grows at single-cell dimensions, and chondrocytes dividing and rearranging to either reshape a fully differentiated cartilage, or form an altogether new cartilage in the midst of a resorbing larval one. More generally, the unexpected superimposition of cell behaviors for shape change onto ones for larval growth, and the unprecedented use of cell cycling to produce extremely large and small chondrocytes open up new directions for investigating the development and evolution of skeletal shape and metamorphic ontogenies.

## Supporting information

S1 Fig Views of the branchial basket flow chambers and cartilage skeleton in a mature, NF 58 larva. (a and b) anterior and posterior views into an E-cadherin-labeled basket at the levels of the otic (inner ear) capsule (oc in a, lower arrow in c) and posterior edge of the eye (b, upper arrow in c). Long arrows in a-b indicate the epithelium-lined anterior and posterior filter surfaces of the first partition; short arrows indicate folds of dorsal epithelium; white arrows indicate mucus secreting columnar epithelium. (c) a ventral view of a dissected, alcian blue-stained basket showing the thickened ceratobranchials (cb1-4) that delimit the “gill” slits (gs, as *Xenopus* tadpoles atypically lack gill filaments, the term is a misnomer for this species). (d) a dorsal view of the region outlined in c showing the vertical arrays of ornate processes in the wall of the basket (long arrows) and the large ornate processes around gill slits (short arrows). (e) a close-up of the part of the partition outlined in d showing adjacent rods (arrows) lined with ornate processes that become smaller dorsally towards their tips. Scale bars for a-c and d-e are 2 and 1 mm respectively. The branchial basket arises in embryogeny from four ceratobranchials laterally and two hypobranchials medially. The cartilages fuse to each other and extend dorsally to form walls and two partitions that enclose three flow chambers. Water enters a flow chamber from the mouth cavity, passes through filter surfaces and exits ventrally via the slit between ceratobranchial bases. The walls of the partitions and inner walls of the basket are lined by thin epithelia that define complex filter surfaces (a-b). The filter surfaces are raised as a result of many, small, polygonally shaped, closely spaced ornate processes of cartilage (or “arboresecent growths” [109]) that lift the epithelium off the supporting cartilage (see also Fig 13A–13C). The ornate processes of arches 1 and 4 are arranged in vertical arrays along the inner walls of the branchial basket (long arrows in d). The vertically aligned ornate processes of arches 2 and 3 are supported by larger cartilages that branch off from each of many thin rods that extend in a line along each ceratobranchial base (arrows in e). The anterior and posterior branches of each rod contribute to the filter surfaces of the flow chambers anterior and posterior to it (Fig 13A–13C). We consider each vertical assemblage of ornate processes and its supporting rod or wall cartilage as a highly modified gill raker (see [108] for more specific terminology and [78] for SEM micrographs of the vertical arrays of ornate processes). The ornate processes decrease in size dorsally and the largest, near the ceratobranchial bases, form a meshwork above each gill slit (small arrows in d). The dorsal edges of walls and partitions are capped with a continuous tract of columnar, mucous secreting epithelium (white arrows in b, mse in Fig 13C) that collects food particles from the ornate processes and transports them to the esophagus [51].
(TIF)

S2 Fig Sections through the three cartilages showing transitions and gradients in chondrocyte size. All are resin-embedded, H&E-stained except b, which is BrdU-labeled, and hematoxylin counter-stained; a-d are frontal sections, and e-f are transverse. (a-c) the changes in cell size and arrangement that ceratohyal chondrocytes go through from their onset of chondrification at NF 43 (a) to the peak of larval cell cluster size at NF 64 (b), and to the emergence of new cell clusters at NF 66+ (c). The NF 64 cell clusters appear to be comprised of numerous, similarly sized chondrocytes that have appeared since NF 58 (also Figs 4H and 4M, and 11A, 11B). Cell size in the NF 66+ clusters ranges from that of the smallest NF 43 chondrocytes to just larger than the largest NF 64 chondrocytes. (d) chondrocytes in the proximal lower jaw at the end of metamorphosis (NF 66+) are generally no bigger than NF 43 chondrocytes (a and Fig 3A). (e-f) gradients in chondrocyte size at the tips of ornate processes (e) and rods (f). Scale bars are 100, 50, and 10 μm.
(TIF)

S3 Fig Using CellProfiler to map the spatial distributions of chondrocyte size, shape, and orientation across entire sections of the NF 58/9 ceratohyal. This involved merging multiple 10 X phase photographs into a composite that captured an entire frontal or transverse section of the ceratohyal. (a) frontal and transverse sections for a left ceratohyal; the arrow shows the approximate level of the transverse section. Each composite was then used to create two images, an inverse, gray scale image of chondrocyte outlines (b) and one of white dots on a black background to indicate the locations of the cell nuclei within the chondrocytes (not shown). All cells had to be given a dot regardless of whether the nucleus was visible in the section. Any slide debris in the gray scale image was removed with Photoshop and any incomplete chondrocyte borders were closed to ensure that regions within the cartilage would be treated as separate cells. CellProfiler, which is free open source software [110], was used to process the two images to digitally outline all cells, and calculate the X, Y coordinates of their centers, the lengths of the long and short dimensions of best fitting ellipses, and the angles of the long dimensions. These data were then used to create a map with each cell represented by an ellipse that conveys its general size, shape, and orientation, and is colored to indicate its cell size-shape class (c). Any ellipse with an axis ratio greater than two is considered an artefact of having to treat all irregular spaces within the cartilage as potential cells. Yellow dots were added to show the location of BrdU-labelled nuclei. Only the ceratobranchial met the CellProfiler requirements that a large central portion could be reliably captured in a single section and that cell outlines are largely contiguous due to minimal matrix accumulation. The results from doing this on NF 47, 53 and 58/9 specimens agree with the quantitative and qualitative results already described.
(TIF)

S1 Table BrdU frequency data. (XLSX)

S2 Table BrdU pulse data. (XLSX)

S3 Table Cell features and percent matrix data. (XLSX)

S4 Table DAPI-stained nucleus data. (XLSX)
