# Supplementary figures and images for: The cellular basis of cartilage growth and shape change in larval and metamorphosing Xenopus frogs
Source: PLoS One. 2023 Jan 12;18(1):e0277110. doi: 10.1371/journal.pone.0277110 (PMC9836273; doi:10.1371/journal.pone.0277110)

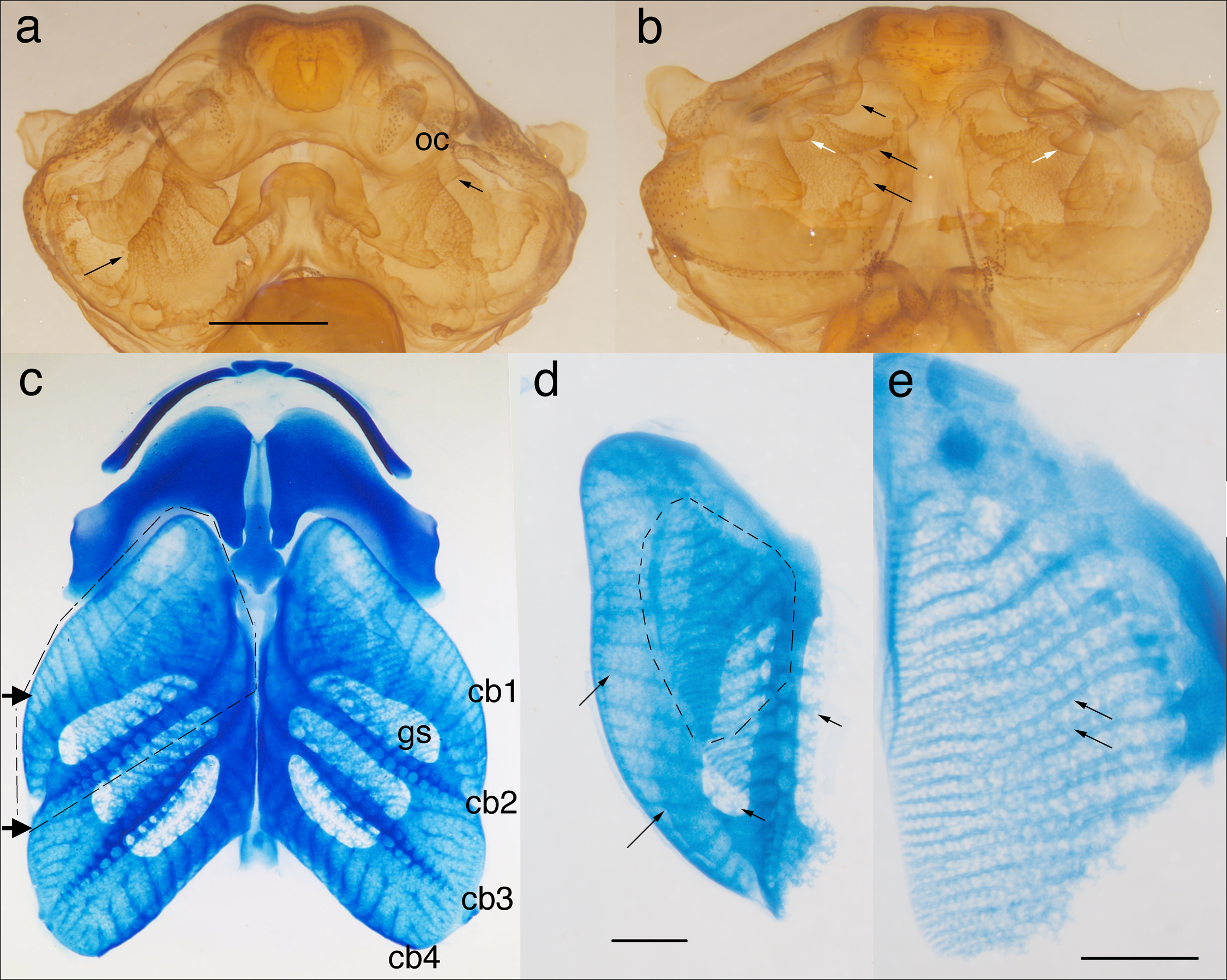

Supplement: S1 Fig — (a and b) anterior and posterior views into an E-cadherin-labeled basket at the levels of the otic (inner ear) capsule (oc in a, lower arrow in c) and posterior edge of the eye (b, upper arrow in c). Long arrows in a-b indicate the epithelium-lined anterior and posterior filter surfaces of the first partition; short arrows indicate folds of dorsal epithelium; white arrows indicate mucus secreting columnar epithelium. (c) a ventral view of a dissected, alcian blue-stained basket showing the thickened ceratobranchials (cb1-4) that delimit the “gill” slits (gs, as Xenopus tadpoles atypically lack gill filaments, the term is a misnomer for this species). (d) a dorsal view of the region outlined in c showing the vertical arrays of ornate processes in the wall of the basket (long arrows) and the large ornate processes around gill slits (short arrows). (e) a close-up of the part of the partition outlined in d showing adjacent rods (arrows) lined with ornate processes that become smaller dorsally towards their tips. Scale bars for a-c and d-e are 2 and 1 mm respectively. The branchial basket arises in embryogeny from four ceratobranchials laterally and two hypobranchials medially. The cartilages fuse to each other and extend dorsally to form walls and two partitions that enclose three flow chambers. Water enters a flow chamber from the mouth cavity, passes through filter surfaces and exits ventrally via the slit between ceratobranchial bases. The walls of the partitions and inner walls of the basket are lined by thin epithelia that define complex filter surfaces (a-b). The filter surfaces are raised as a result of many, small, polygonally shaped, closely spaced ornate processes of cartilage (or “arboresecent growths” [109]) that lift the epithelium off the supporting cartilage (see also Fig 13A–13C). The ornate processes of arches 1 and 4 are arranged in vertical arrays along the inner walls of the branchial basket (long arrows in d). The vertically aligned ornate processes [file pone.0277110.s001.tif]

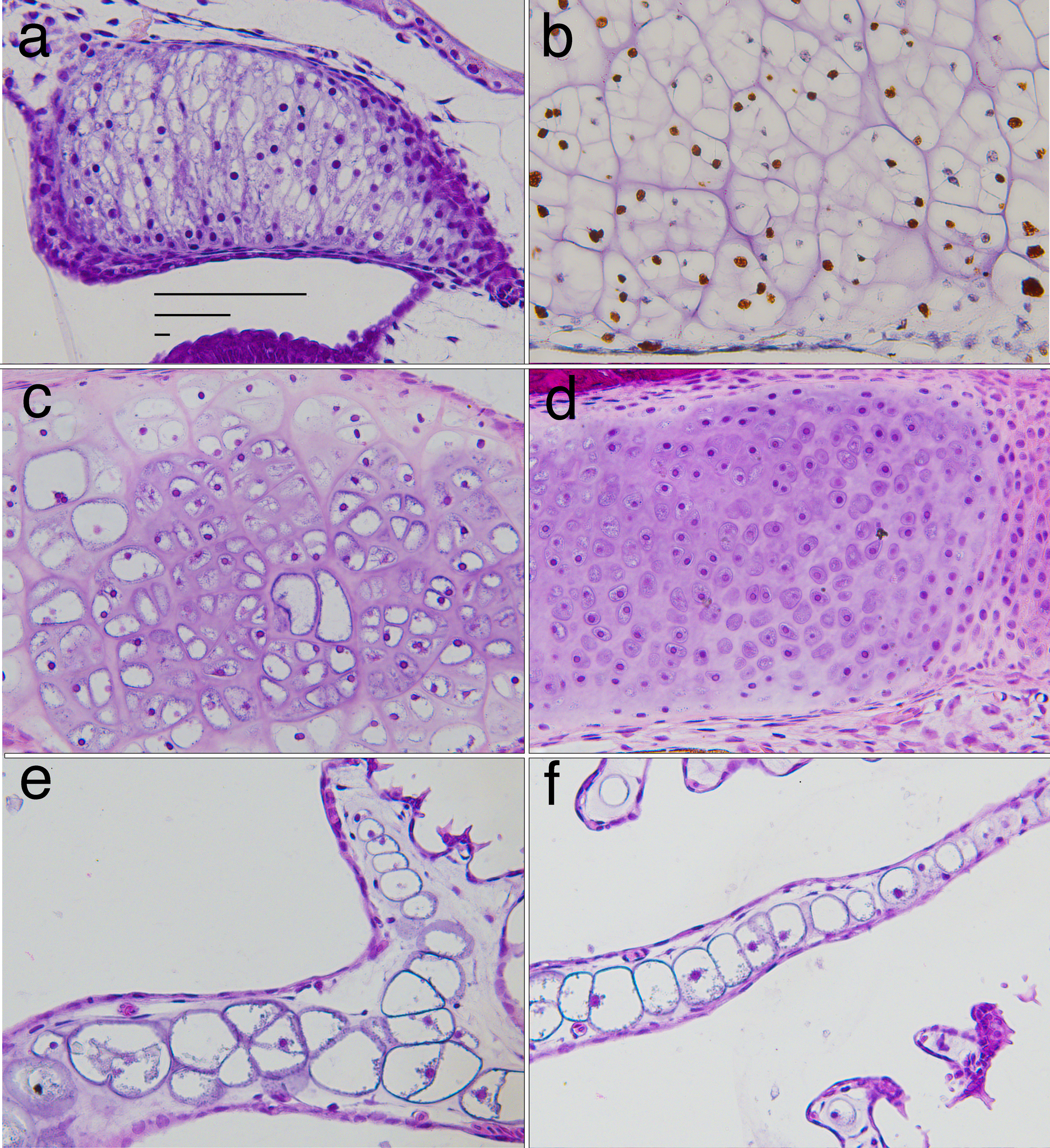

Supplement: S2 Fig — All are resin-embedded, H&E-stained except b, which is BrdU-labeled, and hematoxylin counter-stained; a-d are frontal sections, and e-f are transverse. (a-c) the changes in cell size and arrangement that ceratohyal chondrocytes go through from their onset of chondrification at NF 43 (a) to the peak of larval cell cluster size at NF 64 (b), and to the emergence of new cell clusters at NF 66+ (c). The NF 64 cell clusters appear to be comprised of numerous, similarly sized chondrocytes that have appeared since NF 58 (also Figs 4H and 4M, and 11A, 11B). Cell size in the NF 66+ clusters ranges from that of the smallest NF 43 chondrocytes to just larger than the largest NF 64 chondrocytes. (d) chondrocytes in the proximal lower jaw at the end of metamorphosis (NF 66+) are generally no bigger than NF 43 chondrocytes (a and Fig 3A). (e-f) gradients in chondrocyte size at the tips of ornate processes (e) and rods (f). Scale bars are 100, 50, and 10 μm. (TIF) [file pone.0277110.s002.tif]

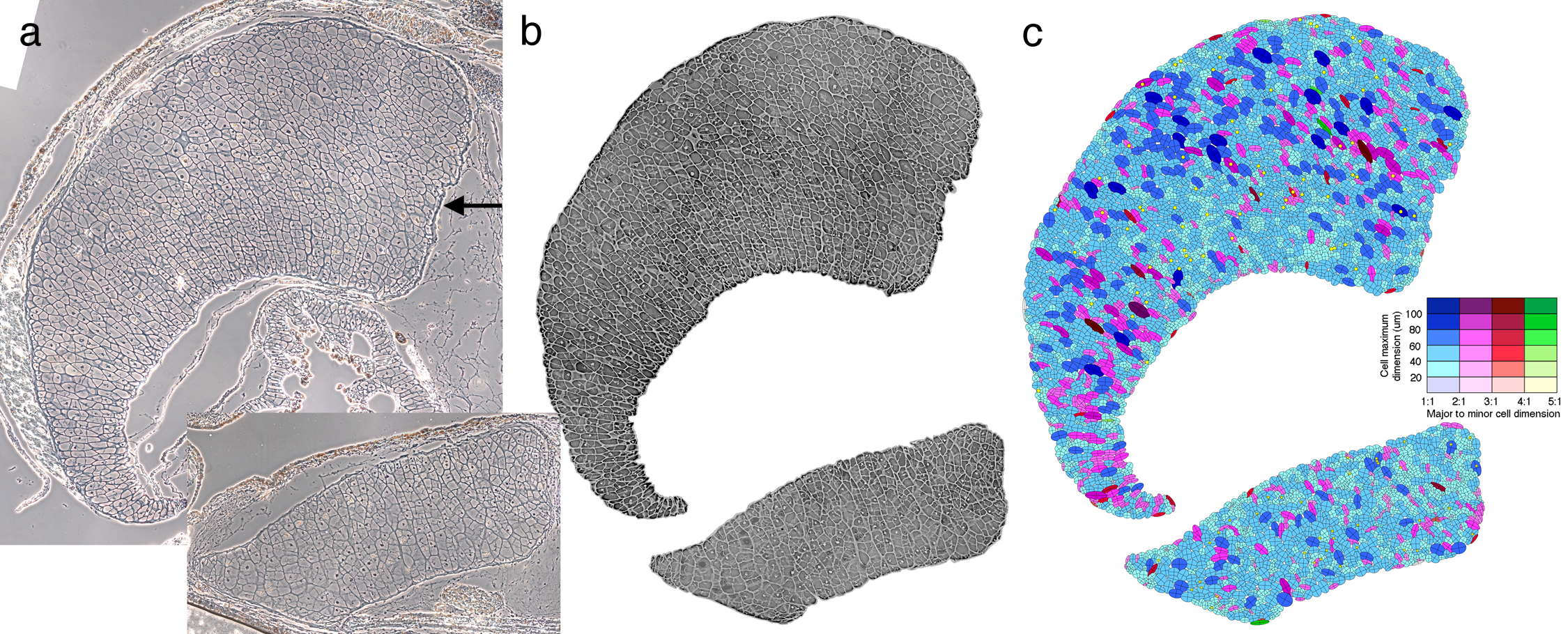

Supplement: S3 Fig — This involved merging multiple 10 X phase photographs into a composite that captured an entire frontal or transverse section of the ceratohyal. (a) frontal and transverse sections for a left ceratohyal; the arrow shows the approximate level of the transverse section. Each composite was then used to create two images, an inverse, gray scale image of chondrocyte outlines (b) and one of white dots on a black background to indicate the locations of the cell nuclei within the chondrocytes (not shown). All cells had to be given a dot regardless of whether the nucleus was visible in the section. Any slide debris in the gray scale image was removed with Photoshop and any incomplete chondrocyte borders were closed to ensure that regions within the cartilage would be treated as separate cells. CellProfiler, which is free open source software [110], was used to process the two images to digitally outline all cells, and calculate the X, Y coordinates of their centers, the lengths of the long and short dimensions of best fitting ellipses, and the angles of the long dimensions. These data were then used to create a map with each cell represented by an ellipse that conveys its general size, shape, and orientation, and is colored to indicate its cell size-shape class (c). Any ellipse with an axis ratio greater than two is considered an artefact of having to treat all irregular spaces within the cartilage as potential cells. Yellow dots were added to show the location of BrdU-labelled nuclei. Only the ceratobranchial met the CellProfiler requirements that a large central portion could be reliably captured in a single section and that cell outlines are largely contiguous due to minimal matrix accumulation. The results from doing this on NF 47, 53 and 58/9 specimens agree with the quantitative and qualitative results already described. (TIF) [file pone.0277110.s003.tif]
